# Blood memory CD8 T cell phenotypes in lung cancer patients predict immune checkpoint treatment responses

**DOI:** 10.3389/fonc.2025.1629802

**Published:** 2025-09-08

**Authors:** Florian Schmidt, Kan Xing Wu, Yovita Ida Purwanti, Nicholas Yan Zhi Tan, Daniel Carbajo, Ke Xin Bok, Andreas Wilm, Michael Fehlings, Daniel MacLeod, Alessandra Nardin, Daniel Tan, Katja Fink

**Affiliations:** ^1^ ImmunoScape Pte. Ltd., Singapore, Singapore; ^2^ National Cancer Centre Singapore, Singapore, Singapore

**Keywords:** immunotherapy, machine learning, cancer immune checkpoint therapy, immuno-oncology, NSCLC, single cell sequence (scRNA-seq), T cell receptor (TCR), cytotoxic T lymphocytes (CTL)

## Abstract

**Background:**

Immune checkpoint inhibition (ICI) has become a standard treatment to re-invigorate tumor-attacking T cell responses in multiple cancer indications, yet a patient’s response is unpredictable even with a confirmed expression of the relevant targets such as PD-1 or PD-L1. Previously identified biomarkers of response have relatively low accuracy, making it difficult to reliably employ them as predictors of clinical response.

**Methods:**

We comprehensively phenotyped peripheral blood CD8+ T cells from patients with non-small cell lung cancer by analyzing surface marker expression, transcriptome, and TCR repertoire with single-cell sequencing technology. The cohorts were comprised of patients who (a) responded to anti-PD(L)1 treatment for a prolonged period of time (b) were new-on-treatment responders, and (c) were new-on-treatment nonresponders. Using various bioinformatics analyses, we defined the signatures of ICI response and evaluated their performance on external scRNA-seq datasets.

**Results:**

We identified response-specific signals in cell type and cell state proportions as well as in TCR repertoire diversity and TCR inter-donor similarity. The enrichment analysis revealed several pathways and regulatory modules enriched in different response groups. Using machine learning, we identified cell-type-specific signatures that predicted the ICI response with an accuracy between 66% and 93% at the single cell level and up to 94% at the patient level. Effector memory CD8+ T cells in long-term responders were most predictive of response, and the inferred effector memory signature could be successfully applied to two related scRNA-seq datasets. CD44, GIMAP4, CD69, and CCL4L2 were among the most relevant contributing markers defining the predictive ML signatures on lung cancer samples.

**Conclusion:**

Our findings suggest that CD8+ T cell subset-specific models reach an accuracy that possesses the potential to inform treatment decisions in a clinical setting.

## Background

T cells are key players in the immune response against tumors but are often attenuated in the tumor environment via intrinsic inhibitory pathways (immune checkpoints). The programmed death 1 (PD-1)/programmed death-ligand 1 (PD-L1) is one of these pathways that mediate the inhibition of T cell activation and proliferation. Targeting the T cell response with immune checkpoint inhibitor (ICI) therapy in the form of monoclonal antibodies against inhibitory T cell receptors has revolutionized the treatment options for cancer patients. ICIs, including anti-PD-1 and anti-PD-L1 antibodies, have become the standard of care for several advance-stage tumor types including non-small cell lung cancer (NSCLC) (1). Some patients may not be eligible for ICI treatment due to either a low expression of inhibitory receptors in tumor biopsies or a low tumor mutational burden (TMB), both of which have been associated with lower response rates (2, 3), yet the expression of inhibitory receptors and/or mutational burden in the tumor do not always correlate with treatment response. Even with a high expression of inhibitory receptors, only a subset of patients responds to ICI treatment (4). At the same time, some PD-L1-negative patients can benefit from ICI therapy (5). Therefore, it is crucial to improve the methods for patient stratification.

Single-cell analysis of immune responses after ICI treatment has increasingly been utilized as a powerful tool to identify molecular differences in responders versus non-responders. Compared to bulk cell analysis methods, the study of single cells enables the discovery of responses that are associated with specific cell subsets and types. This is important because signals in rare cell subsets could be clinically meaningful but might not be visible in bulk cell analysis (6).

A considerable number of studies have analyzed immune cell phenotypes in both tumor tissue and blood after ICI treatment with the aim to identify markers associated with response—for example, in clear cell renal cell carcinoma patients, the response to anti-PD-1 treatment (nivolumab) was associated with a higher expression of CD3E, CD8A, Granzyme B (GZMB), and TCF7 in T cells from responders’ versus non-responders’ tumor samples (7). Based on an analysis using the Cancer Treatment Response gene signature Database (CTR-DB), Hu et al. identified CD69 and SBK1 as potential biomarkers for anti-PD-1/PD-L1 treatment response. A correlation analysis of TCGA data from various cancers showed that CD69 expression correlated positively with immune checkpoint expression and immune infiltration, whereas SBK1 correlated positively or negatively with the same parameters, depending on the cancer type (8). Additionally, the combination of single T cell phenotyping with T cell receptor (TCR) sequence analysis can be informative in the context of post-ICI responses: The expansion of large peripheral T cell clones was associated with durable clinical benefit after ICI treatment in melanoma. A phenotypic analysis showed that these large clones over-expressed cytotoxicity-associated phenotypes (9). Similar observations were reported for NSCLC with the expansion of novel T cell clones early after ICI initiation, whereby the expansion was more extensive in patients with durable clinical benefit. The expanded T cells showed a proliferative or GZMK+ PDCD1+ effector memory CD8+ T cell phenotype (10). In addition, higher TCR diversity was associated with a better outcome in different tumor types (11).

We found previously that NSCLC patients responding to anti-PD-L1 treatment showed high levels of CD57, CD244, and KLRG1 expression on circulating neoantigen-specific T cells (12). CD57 expression in patients before the initiation of treatment was further validated as a possible prognostic marker of a positive response in metastatic urothelial cancer (13). Several additional studies showed that the expression of inhibitory receptors including TIGIT and PD-1 and activation/proliferation markers including HLA-DR, CD38, and Ki67 in blood T cells were associated with the response to ICI treatment early after treatment (14–18). A study on γδ T cells using flow cytometry also found associations between CD3+γδT+CD28− and CD3+PD−1+ cell abundance and response to treatment (19). Separately, an “exhausted” and/or effector T cell phenotype defined by markers including GZMB, perforin, CX3CR1, and interferon gamma (IFN- γ ) expression was repeatedly found to be associated with response (16, 20–23).

In this study, we investigated molecular signatures associated with ICI response using deep phenotypic multi-omics profiles of blood-derived CD8+ T cell samples from NSCLC samples.

## Methods

### Study samples and initial processing

Whole blood was collected in EDTA vacutainer tubes after written informed consent was given by the study participants under Individualized Molecular Profiling for Allocation to Clinical Trials (IMPACT) project (CIRB Ref: 2019/2170). PBMCs were isolated using Ficoll gradient or CPT tubes (Becton Dickinson) and were frozen until further analysis. Slightly less than half of the patients had a high PD-L1 tumor proportion score (TPS) ≥50% (*n* = 7/16, 43.75% two unknown). The patients received either ICI monotherapy or combination treatment with an anti-PD-(L)1 inhibitor and chemotherapy (*n* = 7/16, 43.75% (**Table 1**). The ICIs received in this study included anti-PD-1 inhibitors nivolumab and pembrolizumab and anti-PD-L1 inhibitor atezolizumab.

**Table 1:** Patient information for the Singapore NCCS cohort.

Patient ID	Tumor type	Prior ICI exposure (LTR/NoT)	Overall response (Yes/No)	Tumor proportion score (TPS)	TEM model response prediction	Treatment at response (Single/Combi)	Age	Gender
Long-term responder cohort								
IP1725	Lung	LTR	Yes	0	Yes	Single	70	Male
IP2403	Lung	LTR	Yes	60	Yes	Single	52	Male
IP2634	Lung	LTR	Yes	1	Yes	Combi	51	Male
IP1063	Lung	LTR	Yes	5	Yes	Single	62	Male
IP1202	Lung	LTR	Yes (v1, v2)	100	Yes (v1, v2)	Single	76	Male
IP2577	Lung	LTR	Yes	0.9	Yes	Combi	53	Female
IP1421	Lung	LTR	Yes	75	Yes	Single	67	Male
IP3329	Lung	LTR	Yes	70	Yes	Single	77	Male
IP2903	Lung	LTR	Yes	0	Yes	Combi	51	Female
New-on-treatment (NoT) cohort								
IP2662	Lung	NoT	Yes	NA	Yes	Single	78	Male
IP2542	Lung	NoT	No (v1, v2, v3)	75	No (v1, v2); Yes (v3)	Combi	58	Male
IP2669	Lung	NoT	No (v1, v2)	20	No (v1, v2)	Combi	61	Male
IP2839	Lung	NoT	No (v1, v2)	0	No (v1, v2)	Combi	73	Male

### Single-cell sequencing library preparation

CD8+ T cells were isolated from PBMCs and processed for single-cell RNA sequencing as described in Schmidt et al. (2023). In brief, monocytes and B cells were depleted from PBMCs using CD19+ and CD14+ selection kits (STEMCELL) prior to staining with oligo-tagged, fluorophore-labeled peptide MHC (pMHC) dextramers (10:1 monomer per dextran) (Immudex) for 10 min at room temperature. The cells were then washed prior to staining with anti-CD3 (BioLegend, 300434), anti-CD8 (BioLegend, 344732), anti-CCR7 (BioLegend, 353251), and TotalSeq-C Human Universal Cocktail, V1.0 (BioLegend, 399905). Finally, the stained cells were resuspended in 0.25 μg/mL 7-AAD solution (Biolegend) and sorted via fluorescence-activated cell sorting (FACS) for 7AAD-, dextramer+, CD8+, and CD3+ cells. These cells were topped off with 7AAD-, dextramer-, and CD8+CD3+ cells to achieve a targeted cell recovery of 10,000 cells per donor for single-cell RNA sequencing using Chromium Next GEM Single Cell 5′ Reagent Kits v2 (10X Genomics). Gene expression, V(D)J, and antibody-derived tags (ADT) libraries were prepared according to the kit instructions (10X Genomics, CG000330 Rev E) and sequenced on NovaSeq 6000 (Illumina).

### Computational processing

#### Preprocessing

10X data was processed with CellRanger (version 6.0.1). We used cellranger vdj for VDJ libraries and cellranger count for feature barcode and gene expression libraries. Multiplets were removed using Scrublet (version 0.2.3) (24).

#### Quality control and data normalization

Stringent QC was performed on single-cell level using Seurat (version 4.3.0.1), considering five metrics: (1) the number of detected genes (NODG), (2) the number of unique molecular identifiers (NUMI), (3) the percentage of reads mapped to mitochondrial genes (pMito) or (4) to ribosomal genes (pRibo), as well as (5) the number of detected proteins (NODP) (log10 of the sum of surface marker counts per cell). Thresholds are determined automatically using density calculations contrasting two of the abovementioned metrics in a pairwise manner. This allows a visual interpretation of the density clouds and thresholds. If the pairwise analysis suggests different values for the same metric, the more stringent value is chosen. The chosen thresholds are as follows: NODG: 340–2, 500 NUMI: 500–7, 000 pMitO: 0%–6% pRibo: 18%–55% NODP: >2.75 . Gene expression data was normalized using Seurats Normalize Data function with default parameters. Surface marker data was normalized using DSB (25) with the parameters denoise.counts = TRUE, use.isotype.control = TRUE, and Mouse-IgG1-kappa, Mouse-IgG2a-kappa, Mouse-IgG2b-kappa, Rat-IgG2b-kappa, Rat-IgG1-kappa, Rat-IgG2a-kappa, and ArmHam-IgG as isotype controls.

#### Data integration and annotation

Dimensionality reduction was performed with principal component analysis (PCA). The number of PCs used for further downstream analysis (UMAPs, WNN integration) was determined automatically using the FindElbow function of the DUBStepR (26) (version 1.2.0) package. Seurat’s weighted-nearest-neighbor (WNN) integration technique is used at default parameters (except for the number of PCs, determined by FindElbow) for the multi-modal integration of scRNA-seq and ADT data. T cell subsets and cell states were determined using an in-house panel that was designed largely based on markers described in previous publications (**Supplementary Figure S1**), followed by a manual inspection of the assignment of labels and their markers using RCA2 (27) (version 2.0).

#### Computation of markers associated with response to treatment

To avoid patient-specific biases in detecting differentially expressed genes on the single-cell level (6), we computed treatment response markers in pseudo bulk space, averaging the expression within donors either across all T cell subsets (ALL setting) or within distinct T cell subsets (single cell type setting) using Wilcoxon test. We applied a *p*-value threshold of 0.05 and an absolute minimum log2 fold change of 0.5 for downstream analysis. To avoid biases based on patient-specific TCR repertoires, VDJ gene expression data was excluded at this stage. Pseudo bulk DEGs were computed by comparing two groups (LTR vs. NonR).

#### Activity analysis of transcriptional regulators

Using the python implementation of SCENIC (28) utilizing the database files *allTFs-hg38.txt*, *hg38-10kbp-up-10kbp-down-full-tx-v10-clust.genes-vs-motifs.rankings.feather*, *hg38-10kbp-up-10kbp-down-full-tx-v10-clust.genes-vs-motifs.scores.feather*, *hg38-500bp-up-100bp-down-full-tx-v10-clust.genes-vs-motifs.rankings.feather*, *hg38-500bp-up-100bp-down-full-tx-v10-clust.genes-vs-motifs.scores.feather*, and *motifs-v10nr-clust-nr.hgnc-m0.001-o0.0.tbl*, we obtained individual AUCell scores for all genes and within the LTR and NonR groups. We aggregated those on donor level and computed the fold changes between the LTR and NonR groups to identify differential regulons.

#### Ligand–receptor analysis using NicheNet

To infer cell–cell communication patterns underlying differential gene expression, we performed a NicheNet analysis using the standardized pipeline described in the online vignette (29). NicheNet allows us to study intercellular communication by integrating the expression data of interacting cells with knowledge on ligand-to-target signaling paths to potentially predict ligand–receptor interactions that might drive gene expression changes in cells of interest. We considered the union of DEGs computed above as potential targets. Ligand–receptor predictions were made using NicheNet’s pre-compiled human ligand–target matrix (describing the potential that a ligand may regulate a target gene), ligand–receptor network (with information on potential ligand–receptor bindings), and weighted signaling data (with weights representing the potential that a ligand will bind to a receptor). Here we analyzed cell-to-cell communication in a sub-cell type agnostic fashion by grouping all T cells together and omitting sender-specific distinctions. This provides insights into general signaling trends across the T cell compartment.

#### Enrichment analysis for KEGG, Reactome, and GO terms

Gene Ontology (GO) and pathway enrichment analysis of genes of interest was performed in R, primarily applying tools introduced in the ClusterProfiler (version 4.10.0) and Enrichplot (version 1.22.0) packages, which provide a universal interface for gene functional annotation from a variety of sources and several visualization approaches for interpreting functional enrichment results, respectively. For that purpose, we mapped the gene names to their Entrez IDs using BioMart and the *org.Hs.eg.db* R databases (version 3.18.0). GO defines concepts and classes that describe a gene function and parent–child relationships between concepts (AnnotationDbi version 1.64.1), whereas pathways represent molecular interactions and reaction networks. Over-representation of GO terms and pathways mapped to Entrez IDs is determined by their associated *p*-value calculated by the hypergeometric distribution


$$P(X\geq k)=1-\sum_{i=0}^{k-1}\frac{\left(\begin{array}{c} M\\ i \end{array})(\begin{array}{c} N-M\\ n-i \end{array}\right)}{\left(\begin{array}{c} N\\ n \end{array}\right)}$$

(which corresponds to a one-sided Fisher’s exact test), where *N* is the total number of genes in the background distribution (all human genes), *M* is the number of genes within that distribution that are annotated to the term or pathway, *n* is the size of the list of genes of interest, and *k* is the number of genes within that list which are annotated to the term or pathway. The *p*-values were adjusted for multiple comparisons using the Benjamini–Hochberg procedure, which controls the false discovery rate. An adjusted *p*-value below the 0.05 threshold identifies an over-represented (or enriched) term or pathway.

#### Power analysis using scPower for single-cell data

We used the scPower webserver (30) to perform a power analysis in approximating our dataset. We used the webserver in DE gene mode with the following parameters: organism = *Homo sapiens*, assay = 10x 5’ v2, tissue = blood, cell type = effector memory CD8-positive, cell type frequency = 0.31 (mean of CD8+ effector memory T cell frequency in our data (**Supplementary Table S5**)), sample size ratio = 0.7, reference study = Blueprint (CLL) iCLL-mcLL, total sample size (min) and (max) = 17, cells (min) = 3, 000, cells (max) = 11, 000. Note that the mean number of cells across samples is 5, 800 in our dataset (**Supplementary Table S1**).

#### Power analysis using PROPER for pseudo bulk RNA-seq data

We used the PROPER R-package (31) to perform a power analysis on bulk RNA-seq data to approximate our cell-type-specific pseudo bulk data. We used PROPER with the settings ngenes = 20,000, p.DE = 0.05, lOD = “cheung”, lBaselineExpr = “cheung”, Nreps = c(7,10,13,16,19), Nreps2 = c(10,13,16,19,22), sim.opts = sim.opts.Cheung, DEmethod = “edgeR”, nsims = 20 and executed the summary function with the parameters alpha.type = “fdr”, alpha.nominal = 0.1, stratify.by = “expr”, and delta = 0.5. The thresholds used for the simulation equal the thresholds used for analyzing our pseudo bulk RNA-seq data.

### Inference of T cell signatures that predict ICI response using machine learning

#### Assessing the relevance of confounders

For all available (clinical) metadata (treatment paradigm at response [single/combi], gender, age, tumor proportion score (TPS)) (**Table 1**), we performed $\chi^{2}$ test for categorical and *t*-test for continuous variables to test for the presence of potential confounding effects.

#### Multi-class logistic regression

We used a multi-class logistic regression approach with elastic net penalty implemented in the glmnet (4.1-7) R-package to predict response groups on the single-cell level as carried out previously (6). The elastic net leads to sparse interpretable models by utilizing the grouping effect, i.e., correlated features are kept in the model. This is achieved by the combination of two regularization terms, the ridge and the lasso penalty. The alpha parameter controlling the trade-off between both penalty terms was optimized using a grid search (0.0 to 1.0, with a step size of 0.05) within a leave-one-sample-out (i.e., all data from one donor) cross-validation. A sixfold inner cross-validation using the cv.glmnet procedure was used to find the best lambda parameter on single-cell level. Due to the small sample size, the leave-one-sample-out cross-validation is the only viable cross-validation approach in this application.

#### Feature design

To construct the feature matrix for the classification problem, we considered all genes and surface markers that showed differential expression (log2(foldchange) ≥0.2 and *p*-value <0.1) abundance in the pseudo bulk comparisons described above. We use a lenient cutoff to avoid the exclusion of features that are potentially relevant in combination with other features. The models were trained on both balanced and unbalanced datasets to avoid biases introduced by cell numbers.

#### Feature interpretation to derive biological signatures

Model performance was assessed using a leave-one-sample-out cross-validation and reported as model accuracy. The regression coefficients were integrated across all cross-validation runs to identify features that were robust to changes in the training data. Non-zero regression coefficients of features can be interpreted as potential T cell subset-specific biological signatures linked to patient response.

#### Applications of learned signatures to an independent cohort by Hu et al.

Single-cell RNA sequencing (scRNA-seq) data were obtained from Hu et al. (32). The dataset (GSE207422) was loaded into R (version 4.4.0) as a sparse UMI count matrix, representing the number of unique transcript molecules detected per gene per cell. Patient metadata, including relevant clinical annotations, was integrated into a Seurat object (version 5.2.0). Data normalization was performed using Seurat’s NormalizeData function with default parameters.

Highly variable features (genes with high variance across cells) were identified before performing principal component analysis (PCA). The first 15 principal components (PCs) were selected to construct a shared nearest neighbor (SNN) graph using FindNeighbors, grouping cells based on their similarity in the reduced-dimensional space. Clustering was performed with FindClusters using the Louvain algorithm at a resolution of 0.1, enabling the identification of major cell populations.

Clusters were visualized in two dimensions using uniform manifold approximation and projection (UMAP) (**Supplementary Figure S9**). Major immune cell populations were assigned based on the expression of canonical lineage markers (6). Specifically, B cells were identified by the upregulation of MS4A1, CD19, IgHM, CD69, and FCER; myeloid cells by CD14, CD16, and CGN1; T/NK cells by CD4, CD8A, CD8B, and CD56; and stem/progenitor cells by CD34, CLC, HLF, AREG, MPO, and MME.

A subset of clusters corresponding to T and NK cells, as well as a distinct stem/progenitor cluster, was extracted for further analysis. These cells were re-clustered, and a new UMAP projection was generated. Cluster identities were refined based on gene expression patterns to define CD8+ T cells, naïve and memory T cells, cytotoxic NK cells, and regulatory and effector T cells.

Functional module scores were computed for the T/NK subset using the AddModuleScore function, using the feature genes identified by our models. This approach calculates an enrichment score for our gene sets in each individual cell, facilitating the distinction of the functionally relevant T cell subpopulations defined above. Statistical comparisons of module scores were performed using *t*-tests to identify significant differences between patient groups, specifically major pathologic response (MPR) versus non-major pathologic response (NMPR).

#### Applications of learned signatures to an independent cohort by Kim et al.

Single-cell RNA sequencing (scRNA-seq) data were obtained from Kim et al. (33). The dataset (GSE285888) was loaded into R (version 4.4.0) as a sparse UMI count matrix, representing the number of unique transcript molecules detected per gene per cell. Patient metadata, including relevant clinical annotations, was integrated into a Seurat object (version 5.2.0). Data normalization was performed using Seurat’s NormalizeData function with default parameters.

Highly variable features (genes with high variance across cells) were identified before performing principal component analysis (PCA). The first 20 principal components (PCs) were selected to construct a shared nearest neighbor (SNN) graph using FindNeighbors, grouping the cells based on their similarity in the reduced dimensional space. Clustering was performed with FindClusters using the Louvain algorithm at a resolution of 0.4, enabling the identification of major cell populations using canonical markers (CD3D, CD4, CD8A, CD8B, NCAM1, GNLY, PRF1, GZMB, MS4A1, CD19, CD1C, CLEC10A, JCHAIN, ZMB, ELANE, AZU1, RETN, CD14, FCGR3A, TNFRSF17, XBP1, GYPA, and ALAS2) (6). We identified clusters 0 and 13 to be CD8+ T cells and performed sub-clustering for these clusters (15 pincipal components, 0.5 resolution). T cell subsets were labeled using canonical markers (CD8A, CD8B, CD4, CCR7, SELL, CD62L, LEF1, TCF7, GZMB, PRF1, IFNG, TBX21, IL7R, CD127, GZMK, CX3CR1, KLRG1, PDCD1, LAG3, HAVCR2, TOX, CD69, ITGAE, CD103, CXCR6, FOXP3, IL2RA, CTLA4, IL10, TRGV2, TRDV9, KLRB1, MKI67, PCNA, TOP2A, CENPF, NUSAP1).

Functional module scores were computed as above for three signatures across all T cell subsets as well as for 100 randomly selected cells.

### Clonotype abundance, diversity, and dynamics analysis

Clonotype abundance was calculated across all LTR and NonR clonotypes of the NCCS cohort with respect to response group and T cell subset. Ratios were discretized using the interval borders: 0, l*e*–05, l*e*–04, l*e*–03, 0.01, and 1. Simpson diversity was computed using the diverse R package (version 0.1.5). For clonotype dynamics comparisons across time points, we consider the normalized size of clonotype y in sample x as $NormalisedClonotype_{x}^{y}=\frac{\left|Clonotype_{x}^{y}\right|}{\left|Sample_{x}\right|}$ , where $\left|Sample_{x}\right|$ is the total count of paired TCRs in sample x and $\left|Clonotype_{x}^{y}\right|$ is the TCR count of clonotype y in sample x .

### Data and code availability

Processed data stored in Seurat objects saved in RDS files and code for figure generation and data processing post-quality control are available on Zenodo (10.5281/zenodo.10867209). Supplementary tables are available in an Excel sheet.

## Results

### CD8+ T cell subsets, cell states, and TCR repertoire diversity in ICI responders versus non-responders

**Figure 1:**
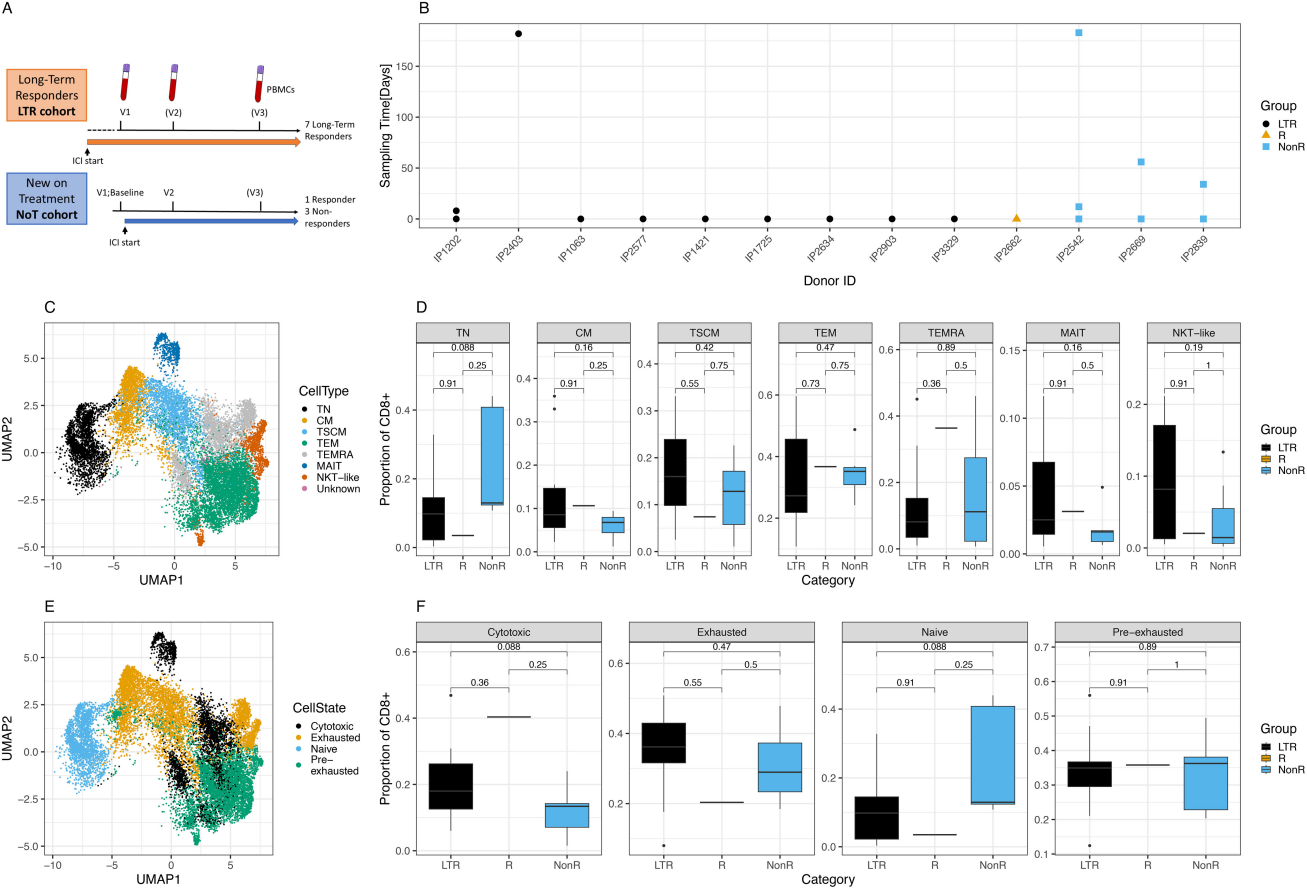
Cohort description and cellular characterization. **(A)** Cohort description and schedule of ICI initiation and blood draws. **(B)** Time points and samples collected for each patient and color-coded response to treatment per patient. LTR, long-term responders; NonR, non-responders. **(C)** UMAP of T cell subsets for all cells analyzed. **(D)** Differences in proportion of CD8+ cells with respect to T cell subsets between response groups. **(E)** UMAP of cell states for all cells analyzed. **(F)** Differences in proportion of CD8+ cells with respect to cell states between response groups.

Tumor-resident or tumor-exposed T cells can circulate and are detectable in the blood (34–36). We analyzed blood-derived CD8+T cells from patients with solid tumor who continue to respond to ICI treatment for a prolonged time, hypothesizing that CD8+ T cells play a key role in these patients to control their tumors. We used single-cell sequencing technology to deeply phenotype these cells and compared data from long-term responders (LTR) to new-on-treatment non-responder (NonR) and responder patients, both at baseline and several weeks after the initiation of treatment. The study cohort from National Cancer Centre Singapore (NCCS) comprises patients distinguished by the timing of ICI treatment initiation: long-term responders (LTR) and new-on-treatment (NoT) patients (**Figure 1A**).

We prospectively recruited LTRs who had previously demonstrated clinical and durable responses to ICI treatment. Among LTRs, the median duration of response was 15 months (range: 4–32 months), with a median duration of follow-up of 21 months. In addition, patients new on ICI treatment were recruited, with blood samples collected immediately before the initiation of ICI and several weeks later. For the NoT patients, the responders were identified from routine clinical and radiological assessment and had achieved at least a partial response after initiation of ICI either alone or in combination with chemotherapy. For some patients, a third sample was collected several months after the ICI start (**Figure 1A**). The time between the first, second, and third sample collection ranged between several days and almost 1 year (**Figure 1B**). Patient details including the tumor indication and responder status for the new-on-treatment group are summarized in **Table 1**.

In total, after quality control, we obtained 104, 400 cells representing data from 10 LTR samples from nine donors, a single sample from a responder patient, and seven NonR samples from three NonR donors.

In a first analysis step, we defined CD8+ T cell subsets and cell states in all patients using scRNA seq data. T cell subsets and cell states were defined based on previously published markers (**Supplementary Figures S1A, B**). T cell subsets, defined by the state of differentiation or maturity, were well delineated and formed mostly uniform clusters in a UMAP illustration (**Figure 1C**). While there were no significant differences in cell type distribution between the two patient groups at the standard 0.05 threshold (**Figure 1D**, **Supplementary Tables S1**-**S6** for cell numbers per patient and metadata), we noticed a trend of naive T cell (TN) depletion in LTRs compared to NonR (Wilcoxon, *p* = 0.088).

Next, we assessed cell states as individual T cell subsets can acquire various cell states describing their function and activation state, which can also be shared across T cell subsets (**Supplementary Figure S1C**). Cells of distinct cell states clustered together in a high-dimensional space except for cytotoxic cells (**Figure 1E**), which formed several clusters, showing more diversity driven by both T cell subset and patient identity. Although significant only at the 0.1 threshold, we found that LTR patients tended to have more cytotoxic cells compared to non-responders (Wilcoxon, *p* = 0.088; **Figure 1F**).

Cells with an exhausted phenotype could be dominated by virus-specific cells, which are known to upregulate exhaustion markers especially during chronic infections (37, 38). To address this, we first used our previously described models to identify CMV, EBV, and influenza (flu)-specific CD8+ T cells based on their phenotype in all patients (39). Inference of viral specificity increased the number of cells for the analysis and included patients with HLA types that were not covered by the dextramer-based detection of viral T cells (**Supplementary Figure S2A**). The predicted specificity matched the dextramer-based reference specificity for most cells (**Supplementary Figure S2B**). The specificity inference resulted in thousands of predicted virus-specific cells (**Supplementary Figure S2C**) that grouped as expected in a phenotype UMAP (**Supplementary Figure S2D**), and the T cell subset and cell state distribution of virus-specific cells matched the previously described phenotypes, notably the TEMRA and exhausted phenotype of CMV-specific cells (**Supplementary Figures S2E, F**) (39). We note a pronounced, but not significant, difference in cell state proportions for CMV-specific cells between LTRs and non-responders, with the latter showing higher numbers of exhausted T cells (Wilcoxon, *p* = 0.055).

Furthermore, we investigated the TCR repertoire of the NCCS cohort in LTR (*n* = 10) and NonR (*n* = 7) samples across all time points: We observed that the clone sizes of unique paired TCRs increased with maturity of the T cell subtypes (**Supplementary Figure S3A**). Furthermore, the Simpson Index revealed that LTR samples had a more diverse set of clonotypes (i.e., more unique TCRs) within the cytotoxic TEM and TEMRA populations compared to NonR samples (**Supplementary Figure S3B**).

In addition, we investigated the across-group TCR similarity by comparing the mean TCRDist similarity across all possible comparisons between samples (excluding same donor but different time-point comparisons). We hypothesized that if the ICI response is indeed driven by tumor-specific T cell, the TCR space of LTRs should be more similar within the LTR group itself than toward NonR as well as NonR compared among each other. As shown in **Supplementary Figure S3C**, this is indeed the case.

Using two time point samples from a long-term responder (patient IP1202), we performed TCR dynamics analyses for paired TCRs (**Supplementary Figure S4**). The second sample (v2), obtained 8 days after the initial sample (v1), harbors many additional clonotypes not present in (v1) (**Supplementary Figure S4A**). Among the 302 shared clonotypes, 250 are expanded in the v2 sample compared to the v1 sample. Unlike the unique clonotypes, for which the largest clone at v2 is of size 8 (**Supplementary Figure S4C**), we observe highly expanded clones among the shared clonotypes in the v2 sample (**Supplementary Figure S4D**)—for example, the largest clone of size 536 is encompassing 12.73% of the entire v2 sample’s TCR space.

### Identification of phenotypic differences between long-term responder and non-responder T cells

**Figure 2:**
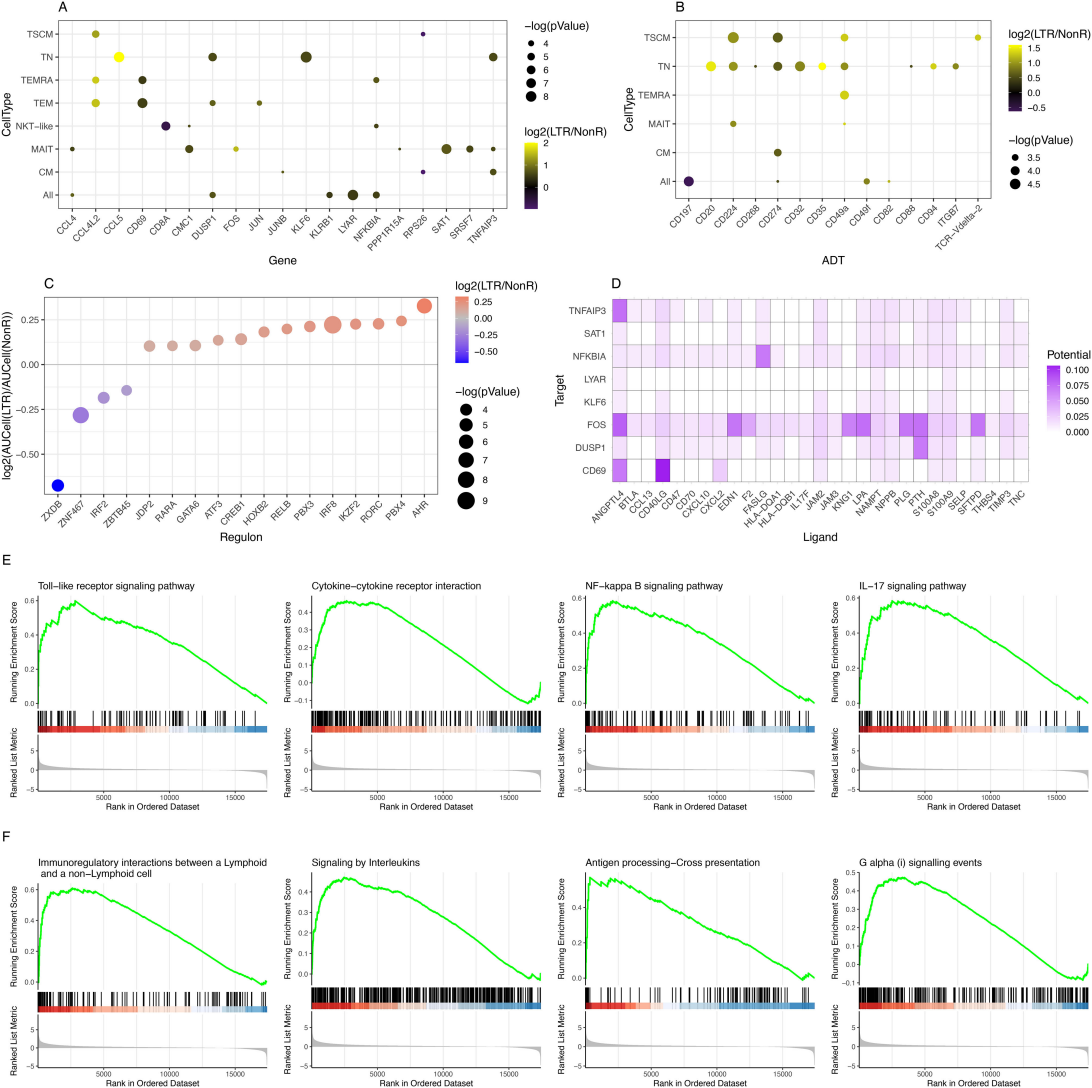
Identification of differentially expressed genes, differentially abundant surface markers, KEGG pathways, and regulons. **(A)** Summary of DEGs in RNA-seq data comparing LTR vs. NonR. **(B)** Summary of differentially abundant surface markers comparing LTR vs. NonR. [For **(A, B)**, a *p*-value threshold of 0.05 and a minimum absolute log2-foldchange of 0.5 are required for a gene/marker to be shown.] **(C)** Bubble plot depicting regulons with differential activity between LTR and NonR samples. **(D)** Enriched ligand–receptor interactions as derived by NicheNet. **(E)** KEGG terms showing enrichment in LTR over NonR samples using pseudo bulked scRNA-seq data as input for a GSEA analysis. **(F)** Reactome terms showing enrichment in LTR over NonR samples using pseudo bulked scRNA-seq data as input for a GSEA analysis.

To study differences in molecular markers for the individual T cell subsets assessed, we next compared differentially expressed genes (DEG) between LTR and NonR. Despite the limited size of our cohort, a power analysis using scPower (30) estimated a DE power of 0.764 for the scRNA-seq fraction of our dataset, suggesting sufficient power to identify DE genes in single-cell space (**Supplementary Figure S5**). However, to avoid bias introduced by unbalanced cell numbers, patient-specific T cell expansions which can occur in scRNA-seq data from cancer samples (6), we performed pseudo bulking per sample across all and within each T cell subpopulation and performed differential expression/abundance calculations in the pseudo bulk space (**Supplementary Table S7**). Approximating power for the pseudo bulk comparisons with the PROPER package (31) resulted in a power estimate close to the suggested threshold of 0.8 (**Supplementary Figure S6**). Due to the nature of the reference data used for the estimate (lymphoblastoid cell lines from 41 unrelated CEU individuals (HapMap) with high biological variation across samples), the power estimation is likely a lower-bound. In our cell-type-specific pseudo bulk data, we found significant positive fold changes for various DEGs, including the CCR5-binding chemo-attractant CCL5 in naive T cells. CCR5-binding chemo-attractant CCL4L2, CD69, DUSP1, NFKBIA, and TNFAIP3 were differentially expressed in several subpopulations (**Figure 2A**).

Separately, we also assessed differences in surface marker expression based on ADT data. We observed an upregulation of the immune checkpoint inhibitors CD224 and CD274 (PD-L1) in TN and TSCM populations, CD35 in TN, and tissue-residency marker CD49a (40) in TN, TSCM, and TEMRA. CD197 (CCR7) was significantly down-regulated in a pan-T cell analysis (**Figure 2B**). CD20, which was upregulated in the TN population (**Figure 2B**), is an established B cell marker that is not known to be expressed in T cells (41). Future studies are needed to elucidate the role of CD20 in the T cell context.

Due to the absence of single-cell epigenomics profiling in our dataset, we next turned to SCENIC (28) to identify potentially relevant regulators that may be associated to gene expression differences between long-term responders and non-responders. As shown in **Figure 2C**, we found 17 transcription factors (TFs) with a significant (*p*-value <0.05) absolute AUCell log2 fold change of at least 0.25 between LTRs and NonRs. We found literature evidence for 15 of these 17 factors to be relevant to T cell regulation (**Supplementary Table S8**). Furthermore, the pseudo bulk gene expression of several of the TFs was in line with the predicted TF activity in either LTRs or NonRs (**Supplementary Figure S7**). Among the TFs associated with the non-responder group was IRF2, which is known to have a feedback loop function redirecting IFN signals, thereby suppressing T cell responses (42). Our analysis also highlighted IRF8 to be associated with LTRs. IRF8 is known to combine TCR stimulation and $\gamma_{c}$ -cytokine signaling pathways. Of particular interest in the lung cancer context, Miyagawa et al. suggested that IRF8 is an essential player in the chronic activation of CD8+ T cells (43). The TF with the strongest enrichment in LTRs was AHR, which is reported to promote a tissue-resident memory gene program. Furthermore, upon AHR depletion, the anti-tumor immunity of polyfunctional CD8+ T cells is diminished (44), which further strengthens the importance of AHR.

A cell–cell communication analysis using the differentially expressed genes and markers as input for NICHNET (29) revealed several ligand–receptor interactions (**Figure 2D**) such as CXCL2 targeting CD69, a tissue residency marker, and FASLG targeting NFKBIA, which codes for a protein inhibiting NF-kappa-B (NF-kB) signaling, thereby regulating immune response (45).

To assess in a more holistic way how LTRs differ from NonR, we performed Gene Set Enrichment Analysis (GSEA) on gene sets from KEGG (46), Reactome (47), and GO (48) using Clusterprofiler (49). In total, we found 11 enriched pathways in KEGG, 11 significant terms in Reactome, and 223 enriched GO terms when applying a *p*.adjust threshold of 0.05 (**Supplementary Tables S9**-**S11**). Based on the DEG, surface markers, and regulons that differ between LTRs and NonRs, we found the KEGG terms *Toll-like receptor signaling pathway*, *cytokine–cytokine receptor interaction*, *NF-kappa B signaling pathway* (linking back to the NICHNET analysis suggesting several potential ligands targeting NFKBIA), and *IL-17 signaling pathway* to be of particular interest (**Figure 2E**).

The Reactome-based analysis also highlighted several pathways linked to the adaptive immune response such as *immunoregulatory interactions between a lymphoid and a non-lymphoid cell*, *signaling by interleukins*, *antigen processing–cross presentation*, and *G alpha (i) signaling events* (**Figure 2F**).

The Biological Process terms identified using the GO analysis listed several processes involved in immune response and signaling such as *GO:0070371*, *ERK1*, and *ERK2 cascade* and *GO:0043410*, *positive regulation of MAPK cascade* complementing the KEGG and Reactome terms (**Supplementary Table S11**).

### Training and validation of machine learning models for the prediction of responses to ICI in LTR and non-responder patients

Based on the various differentially expressed genes and differentially abundant surface markers between LTRs and non-responders, we next sought to identify biomarker signatures that could potentially be useful to predict clinical responses. We used statistical tests to determine whether any potentially confounding covariates from the recorded metadata (**Table 1**) should be included in a machine learning model. We found neither gender ( $\chi^{2}$ test, *p* = 0.21), tumor proportion score (TPS) (*t*-test, *p* =0.87), nor age (*t*-test, *p* = 0.76) to be associated to response—for the association between treatment regimen at response and response itself, $\chi^{2}$ test did show a significant association (*p* = 0.004). However, we decided not to include the treatment regimen at response as a feature to allow for both the incorporation and applicability of the model to new-on-treatment baseline samples.

**Figure 3:**
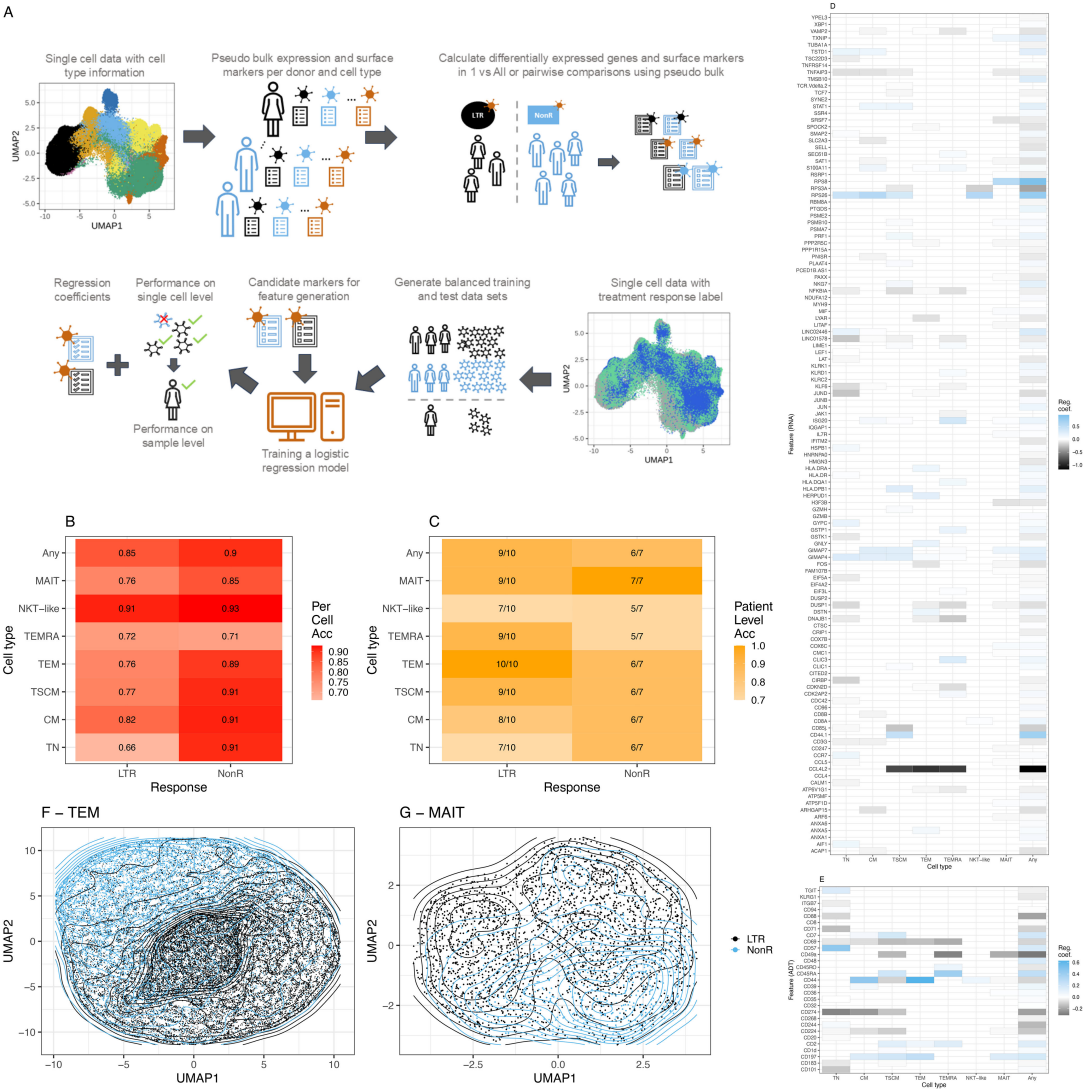
Machine learning model for ICI response prediction. **(A)** Description of the machine learning workflow. **(B)** Balanced accuracy averaged across all leave-one-sample-out cross-validation runs on a single-cell level. **(C)** Model performance on sample level across all leave-one-sample-out cross-validation runs. **(D)** RNA features with a non-zero median regression coefficient across the leave-one-sample-out cross-validation for the two groups, lung-only model. Blue (values closer to 1), marker associated with prediction of NonR; black (values closer to -1), marker associated with the prediction of LTR, whereby 1 and –1 indicate the most relevant features. **(E)** As **(D)** using ADT data. **(F)** Grouping of TEM cells in UMAP space using non-zero features for dimensionality reduction. **(G)** Grouping of MAIT-like cells in UMAP space using non-zero features for dimensionality reduction. Acc = Accuracy.

Given the relative stability of T cell subset and state distribution in longitudinal samples (**Supplementary Figure S8**), we included all patient visits for the training of machine learning models. As illustrated in **Figure 3A**, we used lenient cutoffs on pseudo bulk-derived differentially expressed genes and surface markers ( | log2(foldchange) | ) ≥ 0.2 and *p*-value <0.1) to construct a candidate feature matrix as input for our machine learning models in a T cell subset and comparison-specific manner. Next, we applied data balancing to construct training and test datasets in a leave-one-sample-out cross-validation procedure to assess model performance both on single-cell (**Figure 3B**, **Supplementary Figure S9**) and sample level (**Figure 3C**), whereby we classify a sample to be correctly predicted if more than 50% of single cells have been assigned the correct label. Multiple time points per patient are considered as separate samples in a per-sample evaluation.

In 93% of natural killer T-like (NKT-like) and 91% of TSCM, CM and TN single cells were correctly assigned to belong to a non-responder. We interpret the NKT-like model with caution due to the low frequency of this T cell subset and the consequently small number of cells that was available to train the model. This is the most likely reason for the non-optimal performance of the NKT-like model on sample level. Across all T cell subsets, at sample level, more LTRs were overall correctly predicted compared to NonR. This is likely due to the small sample numbers for the NonR group. A generic model that is trained across all subtypes using the union of differentially expressed genes and surface markers computed on the subtypes obtains the second best accuracy (0.85) in single-cell space for predicting LTRs.

The best results on sample level were observed for the TEM model that predicted all 10 responder samples correctly and for the MAIT model that predicted all seven NonR samples correctly (**Figure 3C**). The mean regression coefficients for each considered feature gene (**Figure 3D**) and each considered surface marker (**Figure 3E**) highlight the T cell subset-specific association of each feature with LTR or NonR status. In general, we observed an agreement of selected features with treatment response between cell-type-specific models and the general T cell model. The separation of cells in a UMAP space based on RNA and ADT features selected by the models is illustrated for TEM and MAIT cells, respectively (**Figures 3F, G**).

In a leave-one-sample-out cross-validation, including a lung responder (R) sample, the TEM model predicted 10 out of 10 LTR, one out of one R, and six out of seven NonR samples correctly (**Supplementary Figure S10A**). Importantly, the analyzed dataset included one baseline sample from responder IP2662 (IP2662-v1), which was predicted correctly as responder even though the model was trained with mostly on-treatment samples.

It is worth noting that the upregulated expression of previously described response surface marker CD57 contributed significantly to the prediction of NonR for TN but not the memory cell models (**Supplementary Figures S10B**, **S11A**). For the TEM model, downregulated CD44 (prediction of NonR (**Supplementary Figures S10B**, **S11A**) and upregulated CCL4L2 [prediction of LTR/R (**Supplementary Figures S10B**, **S11B**)] were the top markers contributing to the model. CCL4L2 is a chemo-attractant for CCR5-expressing cells during inflammation and immune-regulation. This gene has previously been described to be highly upregulated in CD8+ T and NK cells in lung adenocarcinoma tumors (50) and has been associated with favorable disease outcomes (51).

In fact, a literature search of all features with a non-zero regression coefficient in the TEM 2-group model revealed that for 22 of 23 features, prior studies suggest an involvement of the respective genes or surface markers in the T cell immune response or lung cancer biology, respectively (**Supplementary Table S12**)—for instance, GIMAP4, a marker upregulated in non-responders, is known to accelerate T cell death (52). NFKBIA expression was linked to LTRs. It encodes IkappaB α , which is a regulating factor of NFkappaB signaling downstream of the TCR and receptors of the TNF superfamily. Transcription factor NFkappaB is required for cell proliferation and the induction of effector function (53).

Furthermore, DUSP1, linked to LTRs, is known to be relevant for T cell function and activation (54). In addition, our TEM model highlighted factors that have been suggested to be prognostic markers in the context of renal cell carcinomas: LYAR (55) and ATP6V1G1 (23).

In summary, the lung-cancer-trained T cell subset-specific models predicted 17 out of 18 ICI treatment responses from 12 cancer patients correctly. On a per-sample level, the models trained specifically with TEM and MAIT cells showed the best prediction accuracy when applied to the same target T cell subsets.

### Comparison of machine-learning-derived signatures with tumor proportion score and validation with external datasets

#### Tumor proportion score

Clinical assessments of PD-L1, such as combined positive score (CPS) or tumor proportion score (TPS), have been used to identify patients eligible for ICI treatments or to predict their likelihood of response to ICI treatments (56, 57). We have obtained these scores for our cohort (**Table 1**) and compared them to the TEM-specific model predictions. We found that our TEM-specific model was able to accurately identify a non-responder that was otherwise TPS high (*n* = 1) and LTRs that were TPS low (*n* = 5).

#### Response signatures from an anti-LAG-3 and anti-PD-1 combination treatment in patients with melanoma

Huuhtanen et al. performed a scRNA-seq study investigating anti-LAG-3 and anti-PD-1 therapy patients with melanoma by analyzing pretreatment blood samples and blood samples taken 1 and 3 months after therapy from 40 patients. They found a significant expansion of both NK and T cell population in responders. Within the CD8+ T cell population, they found PRF1, NKG7, GNLY, GZMH, MIF, IL7R, and CCL5 to be differentially expressed in responders compared to non-responders (58). These genes have also been identified by our ML model in predicting anti-PD-1 treatment response in lung cancer (**Figure 3D**), suggesting that our phenotypic signature might even be applicable to other indications. Furthermore, the study by Huuhtanen et al. revealed that the CD8+ T cell population in responders showed a more cytotoxic phenotype. This is also in agreement with the trend that we observed in our study (**Figure 1F**).

#### Immune gene signatures for predicting the durable clinical benefit of anti-PD-1 immunotherapy in patients with non-small cell lung cancer

In a study similar to ours, Hwang et al. performed phenotyping using a panel of 395 immune-related genes for tumor tissues extracted from 21 patients with NSCLC tumors before treatment. By contrasting patients with a durable clinical benefit to those without, they identified the expression of PSMB9 to be highly predictive of the clinical outcome (59). Interestingly, our model identified PSMB10 as highly relevant in predicting LTRs (**Figure 3D**). Both genes code for members of the proteasome B-type family (T1B family), which is essential for the processing of class I MHC peptides (60). In addition to PSMB9, Hwang et al. showed in a NSCLC dataset that immune-related gene expression signatures M1 (CBLB, CCR7, CD27, CD48, FOXO1, FYB, HLA-B, HLA-G, IFIH1, IKZF4, LAMP3, NFKBIA, and SAMHD1) and a peripheral T cell signature (HLA-DOA, GPR18, STAT1) have predictive power for predicting clinical benefit. We found that our model also suggested several genes that are part of these previously suggested signatures: CCR7, CD48, and NKKBIA in the M1 signature as well as STAT1 in the peripheral signature.

#### Tumor microenvironment remodeling after neoadjuvant immunotherapy in non-small cell lung cancer revealed by single-cell RNA sequencing

We obtained scRNA-seq data for about 92, 000 single cells from three pre-treatment and 12 post-treatment patients with non-small cell lung cancer (NSCLC) who received anti-PD-1 treatment (32). The patients were grouped based on major pathologic response (MPR) versus non-MPR. Upon identification of major cell types and immune sub-cell types (**Supplementary Figures S12A–D**), as described previously (6), we investigated the discriminatory potential of our suggested prognostic signatures within this dataset by computing the module score for each immune cell type. Considering the union of all features determined to have predictive power as listed in **Figure 3D**, we saw significant differences in module scores across immune cell types and response groups (**Supplementary Figure S13A**). Importantly, restricting the feature list to only features relevant in the effector memory population, which we predicted to be especially relevant, resulted in significantly different module scores between treatment response groups for effector memory cells in this external NSCLC dataset (**Supplementary Figure S13B**). Additionally, the more comprehensive signature of the general T cell model also led to significantly different module scores between MPR and NMPR across all T cell subsets (**Supplementary Figure S14**).

#### Classification of baseline PBMCs in patients with NSCLC

We retrieved single-cell PBMC sequencing data for 222, 144 cells from dataset GSE285888 (33). Kim et al. predicted both ICI efficacy and immune-related adverse events (irAE) severity in patients with NSCLC from baseline PBMC samples. This is especially interesting as the PBMCs were collected prior to ICI treatment. As the cell typing data was not shared by Kim et al., we performed cell type annotation to identify a total of 35, 437 CD8+T cells (**Supplementary Figures S15A–E**). Using our T cell signatures, we then computed the module scores contrasting the patients’ responses to the ICI treatment: CR (complete response), DR (durable response), PD (progressive disease), and irAE (immune-related adverse events). We found that all three suggested feature sets, including the TEM signature, were able to separate PD and irAE from CR (used as a baseline in comparisons) across most CD8+ T cell subsets, while the module scores computed from randomly selected cells did not lead to meaningful differences between patient groups (**Supplementary Figure S15F**). These findings, using an independent dataset, provided an additional validation of our suggested molecular response signatures. Importantly, as the dataset by Kim et al. consisted exclusively of pre-treatment, baseline samples, the validation here clearly demonstrated the potential for predictive response diagnostics prior to ICI treatment using our molecular signatures.

Overall, we found that our signature aligns with previously reported findings and two external scRNAseq datasets despite limited sample numbers and/or challenging data sampling schemes.

## Discussion

**Figure 4:**
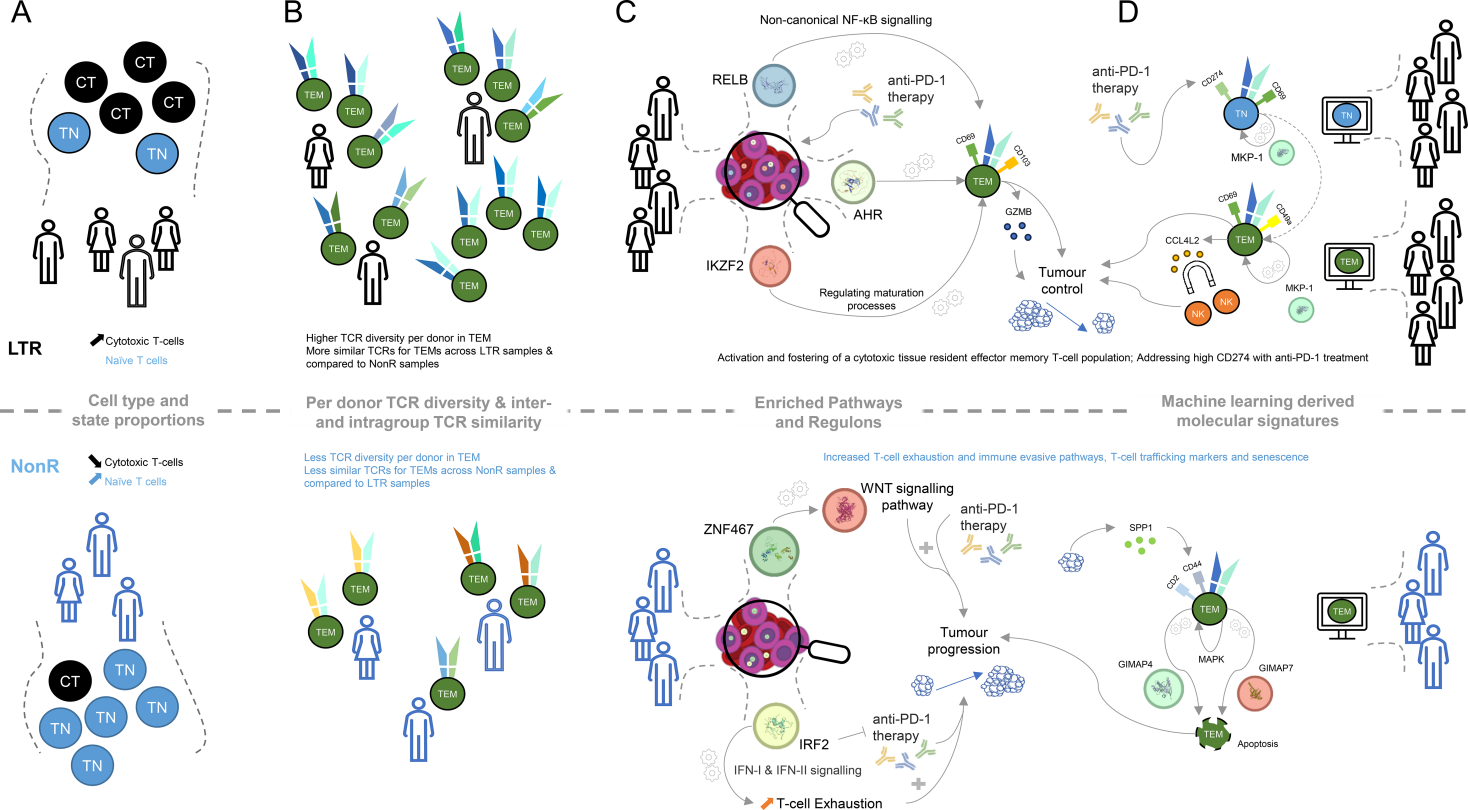
Graphical summary of key molecular differences discovered within lung cancer patients between long-term responders and non-responders. **(A)** Cell type and cell state proportion differences between LTR and NonR (CT, cytotoxic T-cell; TN, naive T-cell). **(B)** TCR diversity and TCR similarity (computed using TCRDist3) difference for effector memory T-cells between LTRs and NonR. **(C)** KEGG pathways and transcription factors determined with SCENIC and their putative linkage to putative links to tumor progression. **(D)** Machine learning-derived genes and surface markers relevant for several cell types and putative links to tumor progression.

In this work, we compared patient groups of LTRs and non-responders to ICI treatment using multi-omics data and various computational methods with the aim to better characterize molecular signatures that are associated to the observed ICI responses. The main findings and hypotheses discussed here are visualized in **Figure 4**.

With respect to cell types and cell states, defined using the CITE-seq data, we observed that the naive T cell population is more abundant in NonR than in LTRs, while cytotoxic T cells are more abundant in LTRs than in NonR (**Figure 4A**). Despite these results being significant only at the 0.1 confidence level, we believe that these differences in cell type proportions and their association to response are relevant as similar associations, across several immune cell types, are reported in another study with a larger cohort size predicting checkpoint inhibitor immunotherapy efficacy (61).

LTRs not only seem to have more cytotoxic T cells but also exhibit a more diverse TCR repertoire (**Figure 4B**). This is in line with a similar observation in a cohort of 12 patients in an attempt to predict durvalumab treatment response in NSCLC (62). Furthermore, our observation that TCRs of LTRs are more similar to each other than to TCRs of NonR or NonR to each other (**Figure 4B**) might suggest the presence of a particular TCR trajectory that is shared among the LTRs but not the non-responders. This could be an interesting area for further investigation. Similarly, exploring TCR dynamics on a dataset with more time point samples generated for long-term responders might be a promising avenue to prioritize TCRs for immunotherapy. Here IP1202 is the only long-term responder for whom we obtained multiple time point samples. We did not perform analyses with the purpose of TCR discovery beyond the ones presented in **Supplementary Figure S4**, but we do believe there is potential for this approach in larger datasets.

While elucidating the gene regulation landscape using SCENIC suggested several transcriptional regulators to be associated with LTRs status, we find RELB, AHR, and IKZF2 to be of particular interest in light of the identified KEGG and REACTOME pathways (**Figure 4C**). RELB is part of the non-canonical NF- κ B pathway (63), which was recently described to be “generating and maintaining CD8+ T cell memory” (53) and could therefore be important for long-lasting tumor control. Similarly, the transcription factor AHR is known to foster a tissue-resident memory CD8 T cell signature that is characterized by CD69 and CD103 positivity and high GZMB production (44). Furthermore, the transcription factor Helios, encoded by IKZF2, is known to orchestrate effector T cell maturation (64). Taken together, these results indicate that LTR patients, when compared to non-responders, maintain CD8+ T cells with a tissue-resident, cytotoxic phenotype.

Among the TFs associated with LTRs, ATF3 has been reported in the context of immune regulation, in particular for regulating NF- κ B expression, cytokine production (65), and anti-tumor activities of T cells (66). For both HOXB2 and GATA6, we did not find any direct evidence associating these transcription factors with T cell regulation. However, the tumor tissue expression of GATA6 has been suggested to identify prognostic signatures in treatment-naive patients with pancreatic cancer (67), making it a prime candidate for further investigation in T cells.

IRF2, which was associated with NonR, has been identified to drive T cell exhaustion and suppressive programs induced by interferons (42). In addition to that, IRF2 deficiency in mice improved anti-PD-1 therapy success, suggesting that upregulation of IRF2 might limit the efficacy of anti-PD-1 therapy in our non-responders (42) and rendering IRF2 a prime candidate for future research.

While we ran the above analyses on the entire T cell population per donor, we next identified DEGs and surface markers in a cell-type-specific way, which enabled the construction of feature gene sets to be used in machine learning to identify complex molecular signatures that predict ICI response.

We identified signatures that may pave the way for further assessment of potential biomarkers of ICI response in patients with lung cancer. Our cell-type-specific models as well as the global model accurately predicted the ICI response in a leave-one-sample-out cross-validation procedure on our in-house dataset. Importantly, we showed that our models were also applicable to external scRNA-seq datasets, showcasing the generalizability of the suggested signatures and thereby encouraging a closer investigation of the relevant features.

The key feature across several cell-type-specific models as well as the global signature that contributed to the LTR model was CCL4L2 (**Figure 4D**). It was positively associated with TEM, TSCM, and TEMRA models in LTR. We speculate that CCL4L2 expression by CD8+ T cells could contribute to the creation of an inflammatory environment by attracting other immune cells, such as NK cells (68). This, in turn, could promote innate mechanisms such as phagocytosis and presentation of tumor antigens. In addition, inflammatory cytokines could help to overcome an immune-suppressive state and re-invigorate T cells. The copy number of CCL4L2 varies between individuals (69), which could be a possible contributing factor influencing the ICI responses and which could partially explain patient-dependent differences in response to ICI treatment.

Similar to the SCENIC and pathway analysis, the machine learning models also pointed us to a tissue-memory-resident signature being indicative of long-term responders. Specifically, CD69, a marker associated with LTRs on TEMs by our machine learning model, is part of the tissue-resident memory T cell signature manifested by the transcription factor AHR. CD69 is a necessary and established marker to control T cell migration, retention, and function (70). We hypothesize that this residency signature is supplemented by CD49A, which was reported in the context of elevated IFN- γ levels upon antigen challenge in melanoma (71). This active state of T cells might be further supported by DUSP1 (MKP-1), which was identified as a positive regulator of T cell activation and cytokine production (54), T cell function, and T cell exhaustion (72). The latter is achieved, for instance, by regulating NFATc1, which is known to regulate T cell cytotoxicity (73). The regulatory potential of DUSP1 (MKP-1) for cytokine production links back to the enriched KEGG term of *cytokine–cytokine receptor interactions*, and the importance of CCL4L2 for our model suggests that proper understanding and future investigation of cytokines in LTRs are of particular interest.

The machine-learning-derived signature also highlights several distinct features on TEMs from NonR. To interpret those, it is important to note that successful anti-PD-1 therapy seems to require a pre-existing anti-tumor T cell response (74). Interestingly, the TEM population described by the ML model does not suggest a strong cytotoxic phenotype. Among the predictive surface markers are CD2 and CD44. CD2 is an adhesion molecule involved in T cell activation and immune synapse formation and has been suggested to be also involved in impacting T cell exhaustion (75). CD44 has been attributed to a plethora of roles in the context of T cells, including T cell migration (in contrast to the tissue-resident phenotype in LTRs), regulation of T cell responses, signal transduction in T cells, and regulation of activation-induced cell death (76). The latter is characterized by the interaction of SPP1-secreting tumor cells and CD44+ CD8+ exhausted T cells activating MAPK signaling (77).

Induced cell death is particularly interesting as we see GIMAP4 and GIMAP7 being selected as prognostic features for the non-responder group as well. GIMAPs are known to be expressed in lymphocytes and regulate survival/death signaling and cell development within the immune system (78). The combination of these markers suggests that their co-expression and/or interaction may exacerbate T cell exhaustion and impair T cell function in the tumor microenvironment, thereby reducing the response to anti-PD-1 treatment.

While more work is needed to elucidate their exact mechanistic roles in T cell response to ICI treatment, several of the predictive features uncovered here have also been reported as potential prognostic biomarkers for response to ICI treatments in varying contexts. GIMAPs, in general, were identified as biomarkers for ICI treatment in lung adenocarcinoma (LUAD), with higher GIMAP expression in tumors associated with therapeutic response (79). GIMAP4 expression in NSCLC tumors was also found to be positively associated with immune checkpoint factors such as PD-L1 and PD-1, while being negatively associated with overall survival (80). GIMAP7 was reported as a pan-cancer biomarker, showing a positive correlation to T cell infiltration of tumors, PD-L1 expression, microsatellite instability, TMB score, and TIDE score (81). CCL4 was identified as part of a four-chemokine expression signature for tumor samples, c-score, that can identify potential response to ICI treatments across several solid cancer types (82). Differing by only one amino acid, CCL4L2 and CCL4 are non-allelic gene copies with shared functions (83). Moutafi et al. identified CD44 as a predictor of ICI response from spatial proteomic profiling of tumors from advanced NSCLC undergoing ICI (84). CD69 expression in lung and breast cancer tumors were positively correlated with immune checkpoint expression, T cell infiltration, and ImmunoPhenoScore (IPS) (85), leading the authors to propose CD69 as predictive biomarkers for ICI treatment response (8). Finally, even as ICI treatments were largely ineffective in the treatment of advanced pancreatic ductal adenocarcinoma, PD-1 blockade resulted in the reactivation of circulating and tumor-infiltrating T cells as characterized by NF-kB signaling (86). These studies represent a collective recognition of the need for better patient stratification that will allow more patients to benefit from ICI treatments. Supporting this notion toward a biomarker-informed management of ICI treatments, a phase 2 HUDSON study attempted a biomarker-driven combinatorial approach for advanced NSCLC patients with primary and acquired resistance to anti-PD(L)1 treatments. In spite of a limited cohort and confounders in disease baselines and demographics, the authors reported an efficacy signal for patients found with a taxia–telangiectasia mutated (ATM) alterations using a combinatorial treatment of durvalumab (anti-PDL1) and ceralasertib, an ATM/ATR (ATM- and Rad3-related) inhibitor (87).

Here we were able to identify these previously reported ICI response-correlated factors as part of our broad molecular signatures. The integrative nature of our machine learning approach is well suited in providing a more nuanced and comprehensive look at the complexities of ICI therapeutic response that otherwise traditional approaches, such as TMB and single biomarker detection, are unable to fully capture. Importantly, while most of the above-cited studies involved working with tumor biopsies, we have accomplished the response-associated markers using data from circulating T cells, a minimally non-invasive source of biomarker sampling. With further refinement, our approach can potentially provide a minimally invasive and more accurate screening method to identify patients who are more likely to respond to ICI treatments.

Just like for cell type proportion differences, the ML models could potentially be improved with a larger sample size with a more consistent timing of sampling. This would allow the identification of potential response signatures both before and during treatment. Computationally, strategies moving forward aiming to enhance the predictive capabilities of our models would be (1) to attempt to train ensemble models, combining the T cell subset-specific predictions into a unifying model and (2) using non-linear models, which, however, due to small sample size, might not be advisable at this stage.

In summary, the strong alignment with previous literature and agreement with external single cell sequencing data provide evidence of the validity and wider applicability of the molecular signatures identified in this study. This study is limited by a small cohort size and its heterogeneity with regard to the time point of blood collection, cancer stage, and type of ICI treatment (**Figure 1**). Future research on a more comprehensive and carefully curated dataset could pave the way to establish flow cytometry or transcriptome sequencing-based assays in clinical practice to informed ICI treatment decisions.

## Data Availability

The datasets presented in this study can be found in online repositories. The names of the repository/repositories and accession number(s) can be found in the article/**Supplementary Material**.
