# Supplementary material for: Blood memory CD8 T cell phenotypes in lung cancer patients predict immune checkpoint treatment responses
Source: Front Oncol. 2025 Sep 8;15:1629802. doi: 10.3389/fonc.2025.1629802 (PMC12452098; doi:10.3389/fonc.2025.1629802)
Supplement: Supplementary file 1 [file DataSheet1.pdf]

Supplementary

Supplementary Tables

All Supplementary Tables are summarized in on Excel sheet available online.

Supplementary Figures

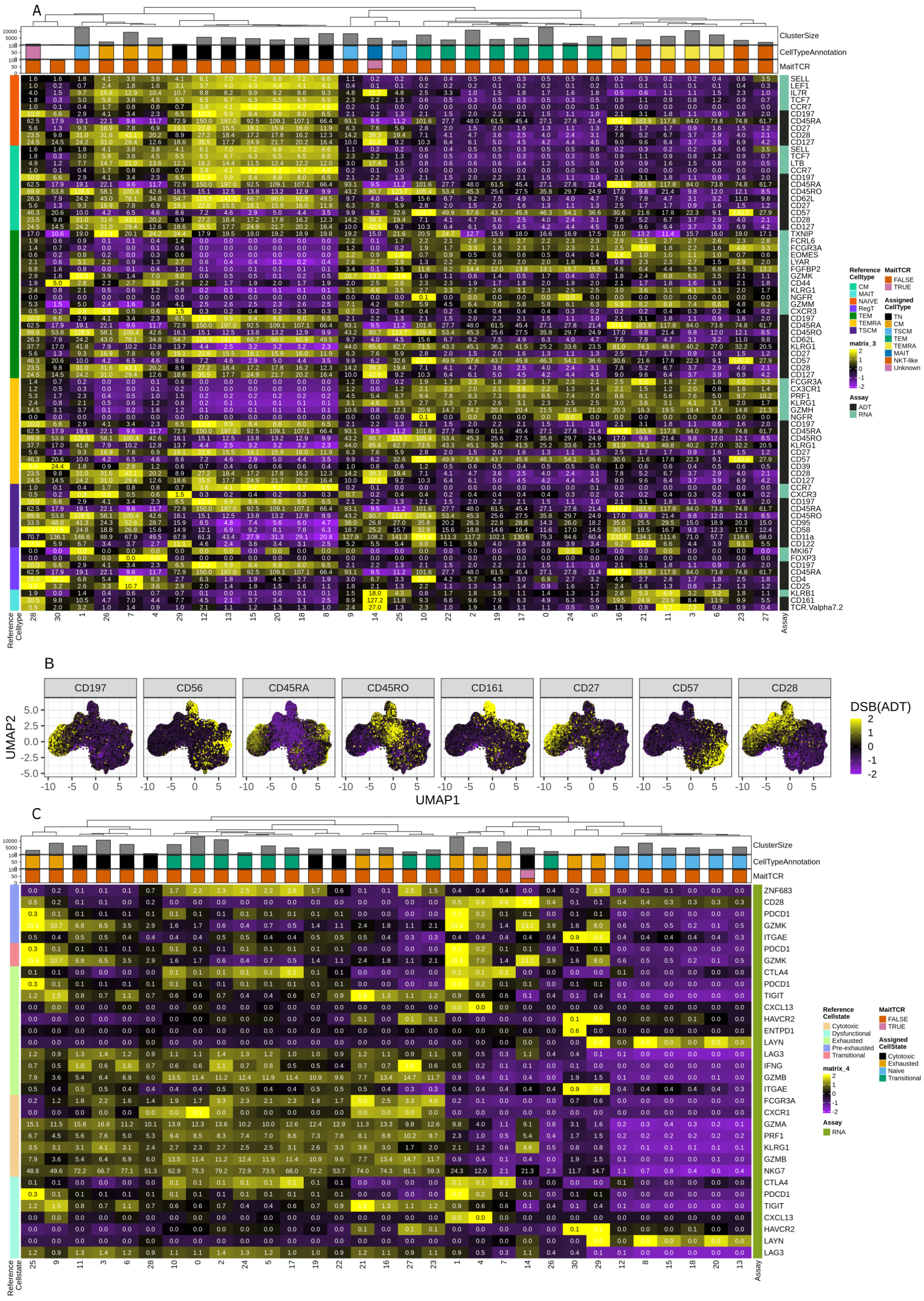

Supplementary Figure S1: **T cell subset and cell state annotation** (Markers based on Kumar BV et al. 2018, https://doi.org/10.1016/j.immuni.2018.01.007; van der Leun AM et al. 2020, https://doi.org/10.1038/s41568-019-0235-4). **A** Markers for T cell subset assignment. **B** UMAP illustration of key surface markers used for T cell subset inference. **C** Reference markers for cell state assignment.

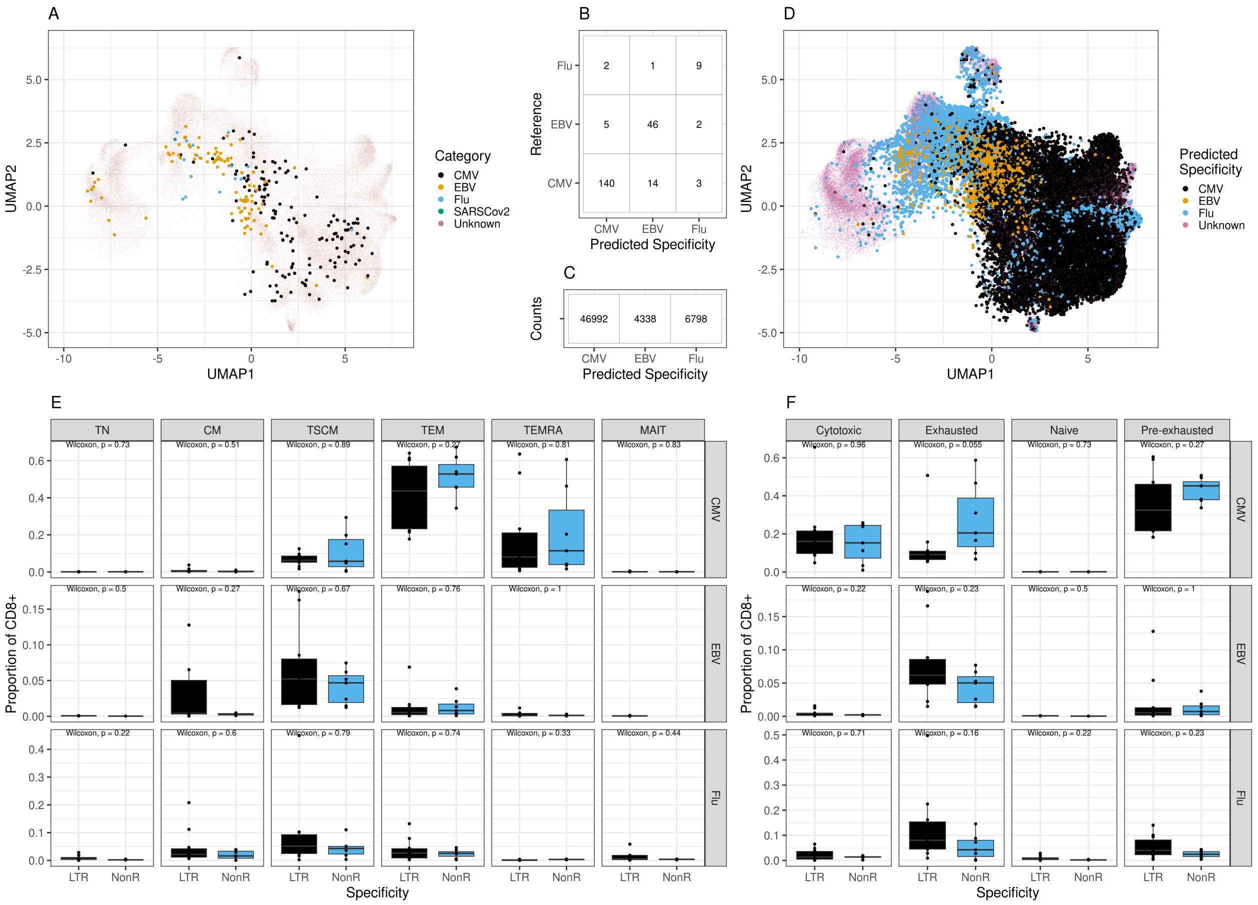

Supplementary Figure S2: **T cell subset and cell state analysis for virus specific cells.** **A** UMAP highlighting cells with experimentally determined viral specificity based on tetramers and mass cytometry analysis. **B** Confusion matrix showing performance of our in-house cell surface phenotype-based ML model to infer viral specificity (Predicted Specificity) on experimentally confirmed T cells with viral specificity (Reference). **C** Number of cells with predicted viral specificity. **D** UMAP highlighting cells with predicted viral specificity for CMV, EBV and Flu. **E** T cell subset-specific proportions of virus-specific CD8+ cells in LTR and NonR patients. **F** Cell state-specific proportions of virus-specific CD8+ cells in LTR, R and NonR patients. **E and F)** Each dot represents o sample.

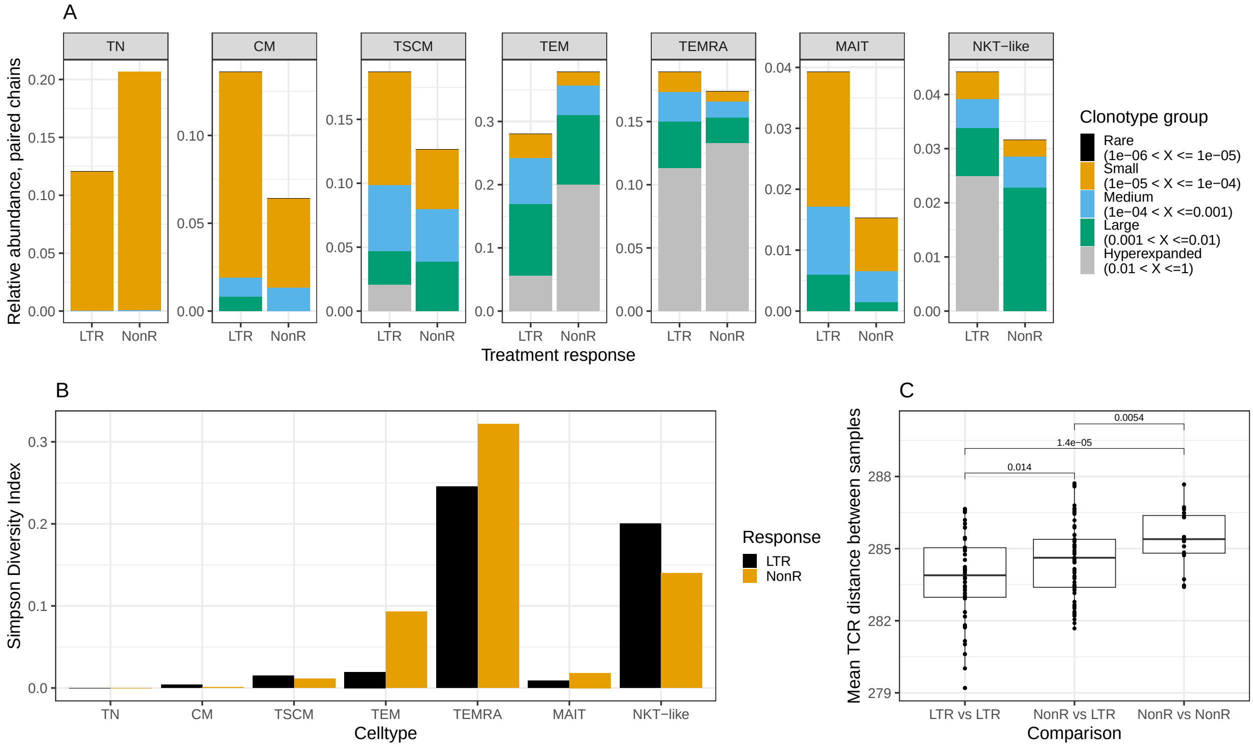

Supplementary Figure S3: **Analysis of clonotype repertoire. A** Relative abundance of clonotypes across T cell subset and per response group. **B** Simpson's diversity index with respect to T cell subset and per response group. High values indicate low diversity, low values indicate high diversity (LTR: n=10 samples, NonR: n=7 samples). **C** Across response group comparisons using per sample TCR similarity computed using TCRDist. A single point represents a comparison between the mean TCRDistance across all TCRs within a sample compared to another one of the same group. Comparisons for different time-points from one donor are excluded.

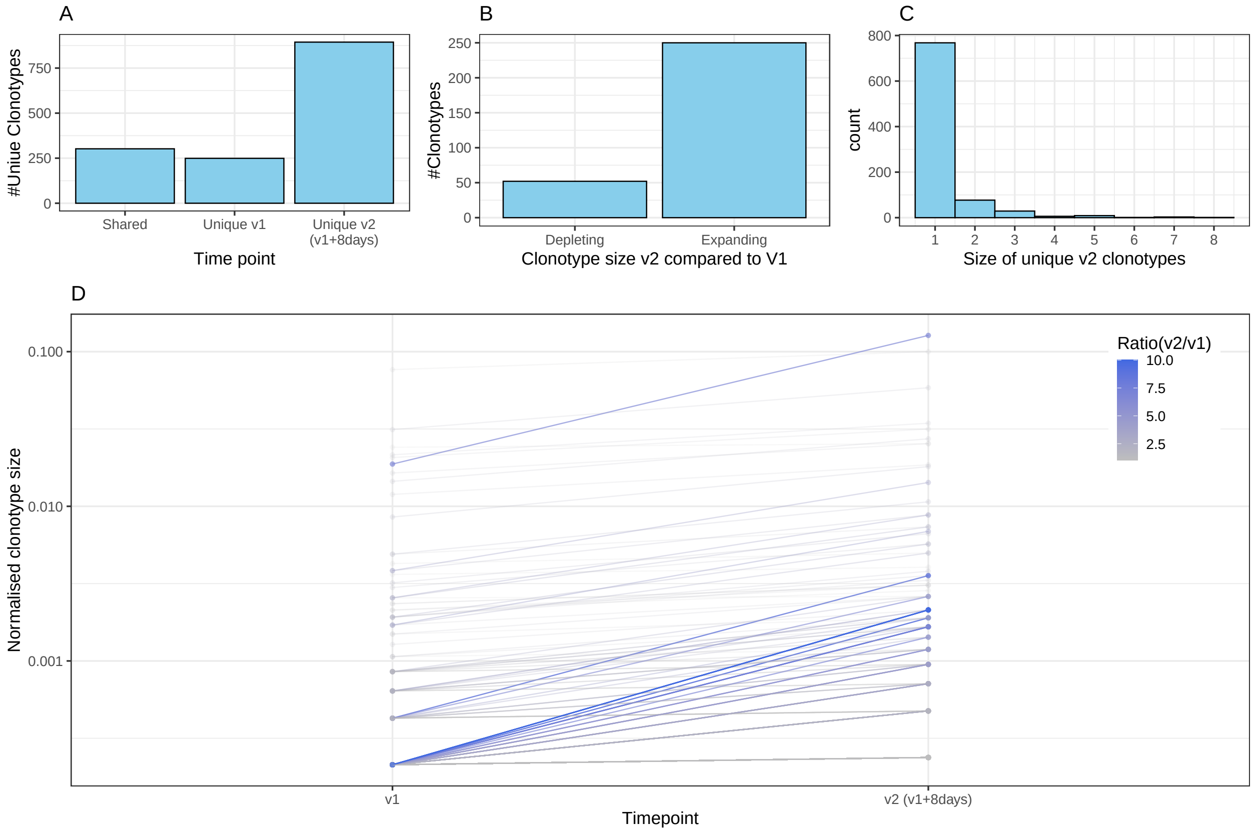

Supplementary Figure S4: **Analysis of clonotype dynamics for two samples from a LTR donor at two timepoints v1 and v2, with v2=v1+8days.** **A** Number of unique clonotypes (y-axis) that are shared among both samples or that are unique for either time point. **B** Number of shared clonotypes that increase or shrink comparing samples from v2 and v1 (comparison accounts for total number of clones per sample). **C** Size of clones that occur only in the v2 sample. **D** Visualization of clonal expansion for shared clones, relative to the total number of clones in each sample.

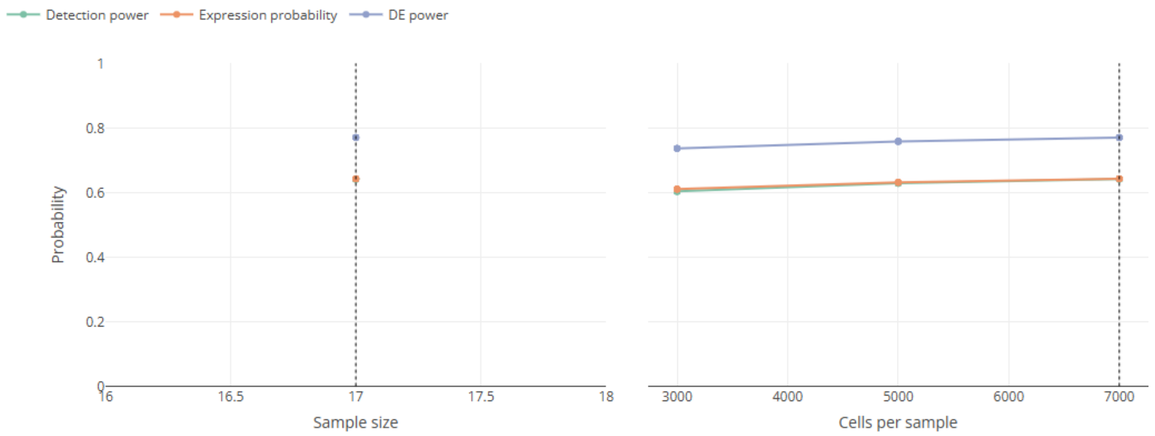

Supplementary Figure S5: **Power analysis performed using scPower [30].** Calculations performed using the parameters Organism=Homo sapiens, Assay=10x 5' v2, Tissue=blood, Cell type= effector memory CD8-positive, Cell type frequency = 0.31 (Mean of CD8+Effector memory T cells frequency in our data (Sup.Tab.5)), Sample size ratio = 0.7, Reference study = Blueprint (CLL) iCLL-mcLL, Total sample size (min) and (max) = 17, cells (min)=3000, Cells(max) = 11000. Our lung cancer cohort has a mean number of cells per sample of 5800, resulting in a DE power value of 0.764.

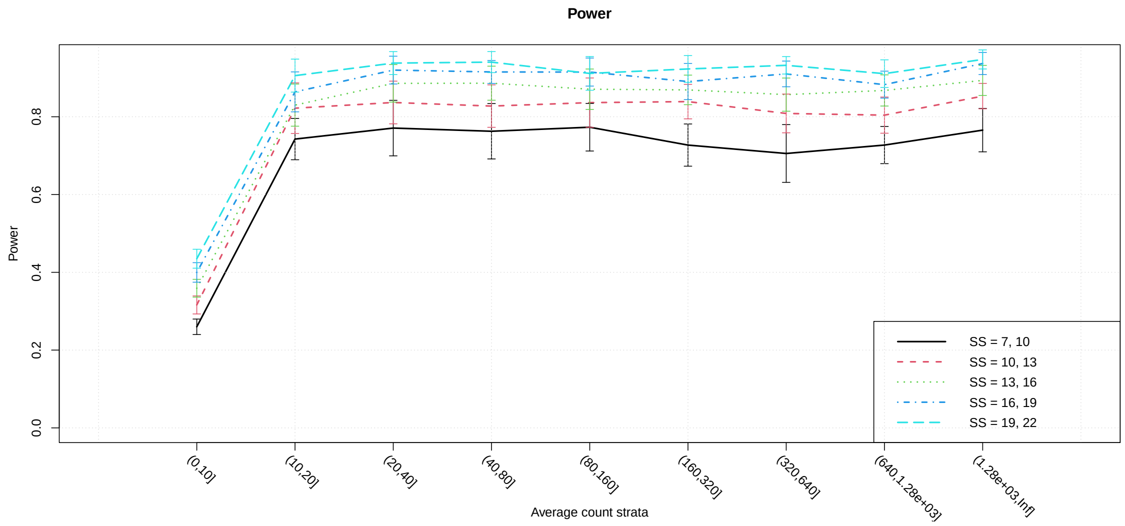

Supplementary Figure S6: **Power analysis performed using the PROPER [31] package (Average reads number per gene on the x-axis, power on the y-axis).** We used the parameters setting ngenes = 20000, p.DE=0.05, lOD="cheung", lBaselineExpr="cheung" and Nreps = c(7,10,13,16,19), Nreps2 = c(10,13,16,19,22), sim.opts=sim.opts.Cheung, DEmethod="edgeR", nsims=20. The estimated power for our cell type specific pseudo bulk RNA-seq data is close to 80% (n=7,n=10).

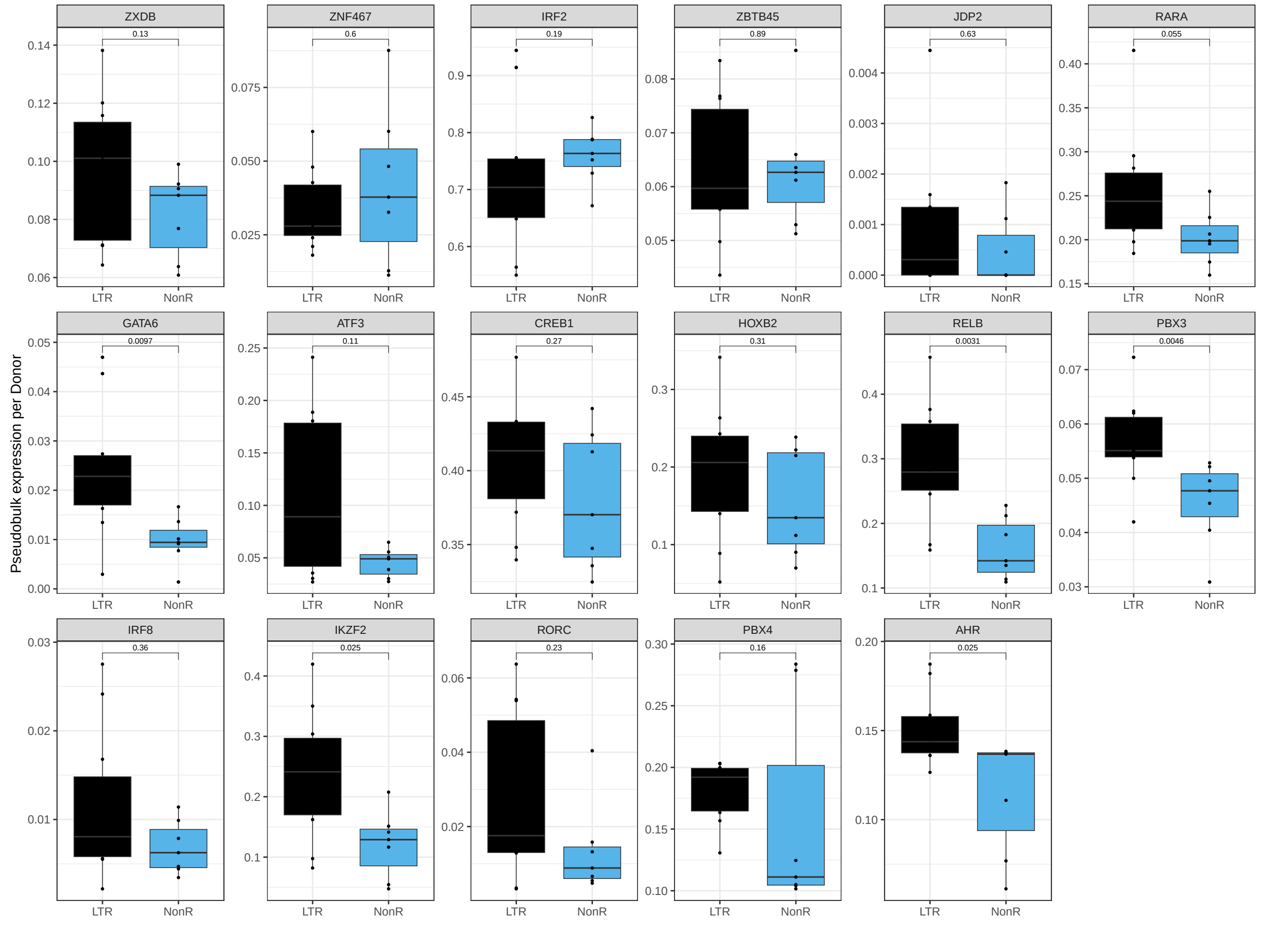

Supplementary Figure S7: **Expression of transcription factors in LTRs and NonRs computed in pseudo bulk space.**

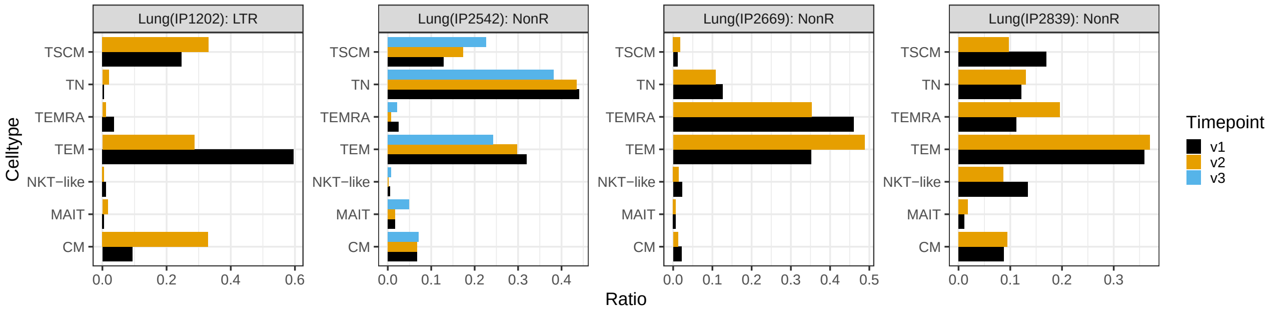

Supplementary Figure S8: **Celltype distributions across time points.**

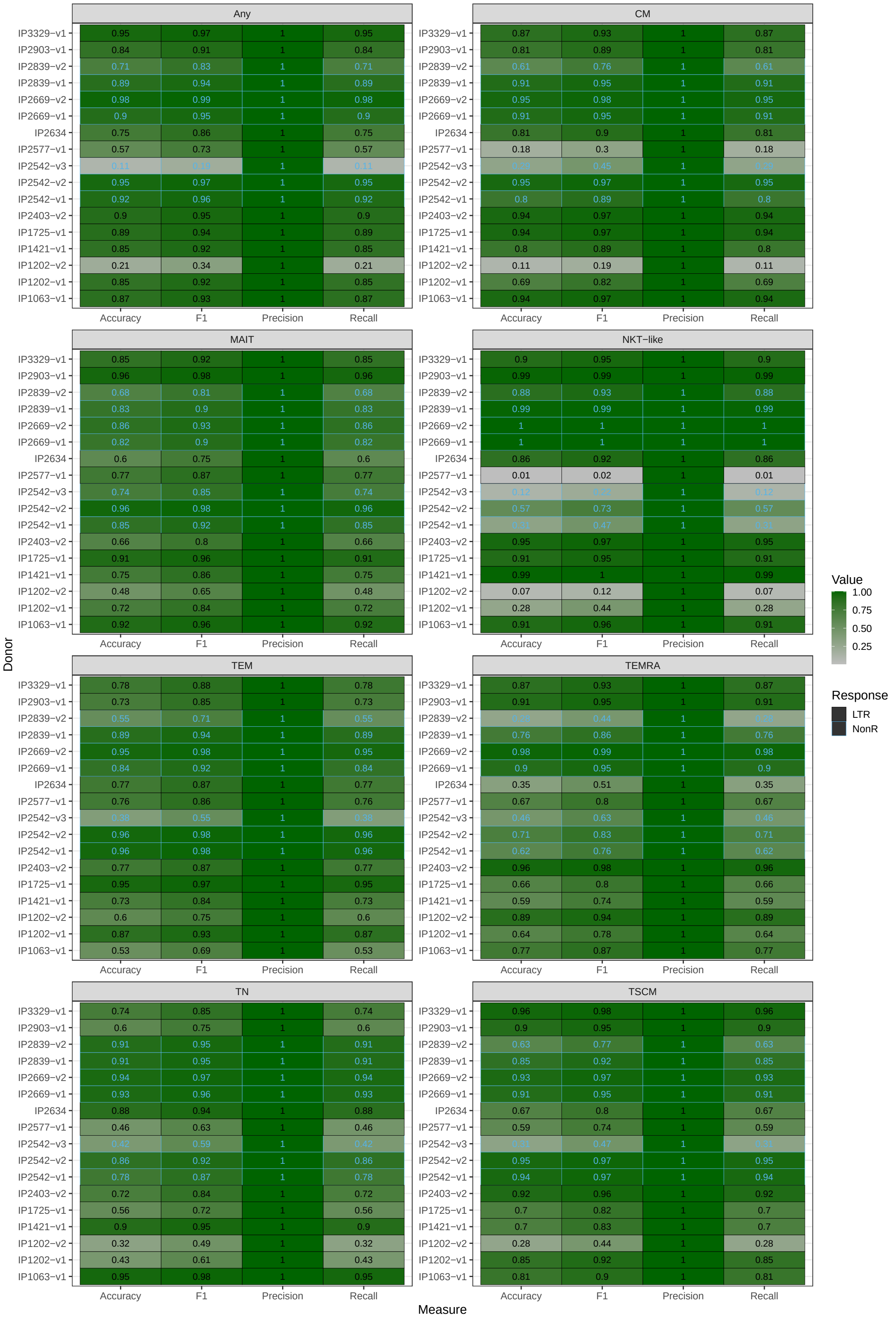

Supplementary Figure S9: **Leave-one-sample out cross validation results per donor.**

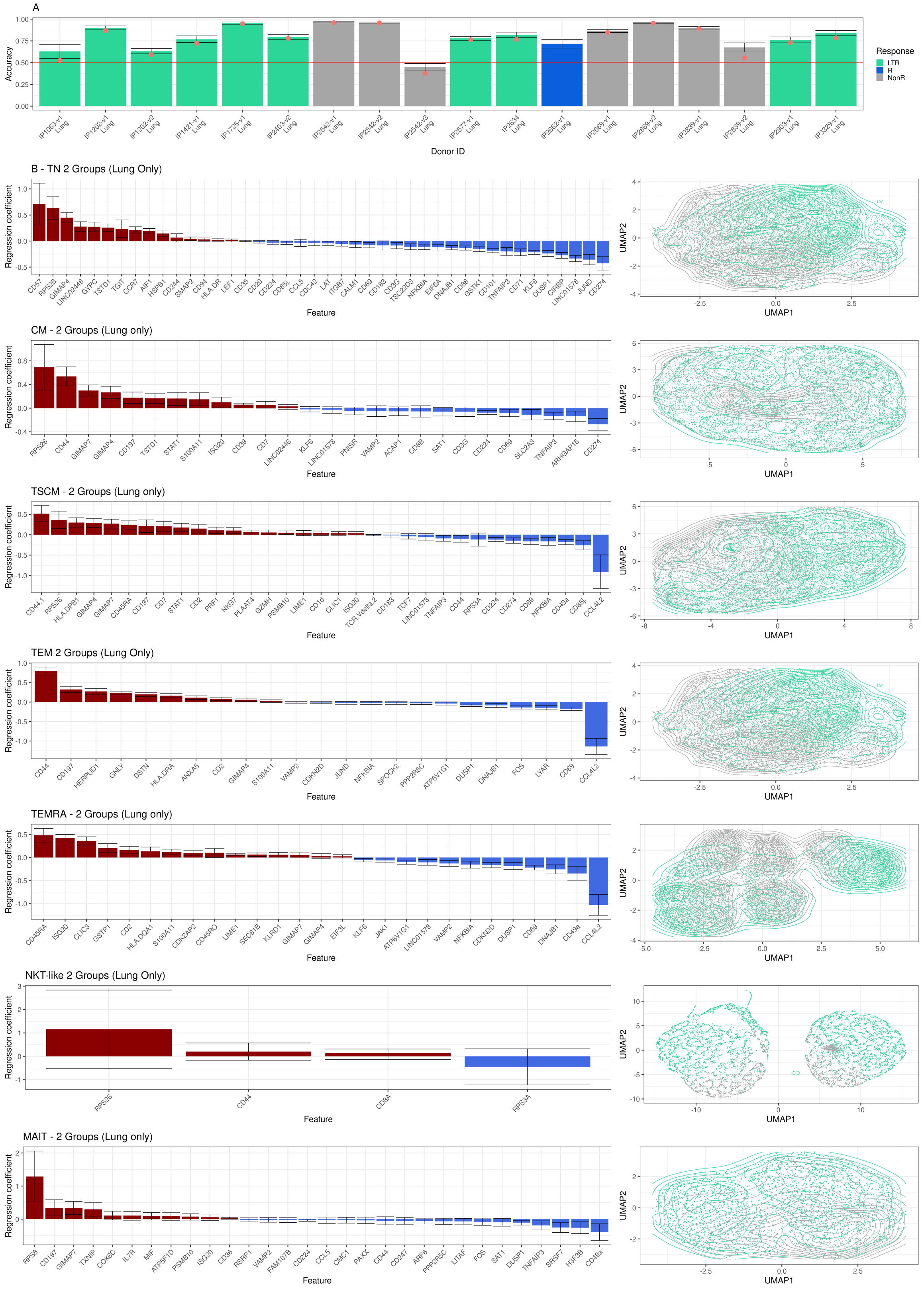

Supplementary Figure S10: **A** Model performance in terms of test error (dots) and training error (bar charts) across all lung samples from the NCCS LTR cohort and test error on one (new on treatment) responder baseline sample (IP2662-v1), applying the 2-group, lung-only TEM model. **B Left panels:** Model coefficients for the 2-groups (LTR vs NonR) lung-only models across T cell subsets; Red (values closer to 1): Association with NonR, Blue (values closer to -1): Association with LTR. **Right panels:** separation in UMAP space using the non-zero features for dimensionality reduction.

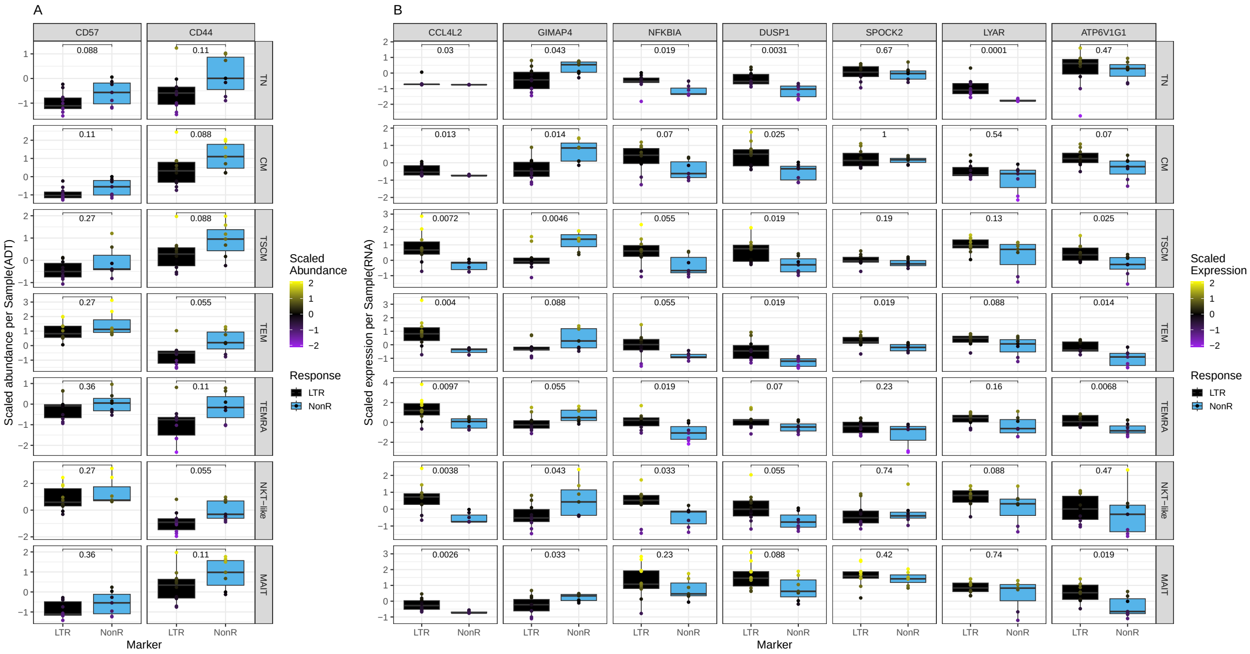

Supplementary Figure S11: **Pseudo bulk abundance/expression of relevant features. A** Scaled abundance per sample for surface markers selected by the predictive models as relevant features. **B** Scaled expression per sample for genes markers selected by the predictive models as relevant features. Significance is assessed with a 2-sided Wilcoxon test. Scaled abundance/expression per patient is capped at 2.1 and -2.1 for color coding only.

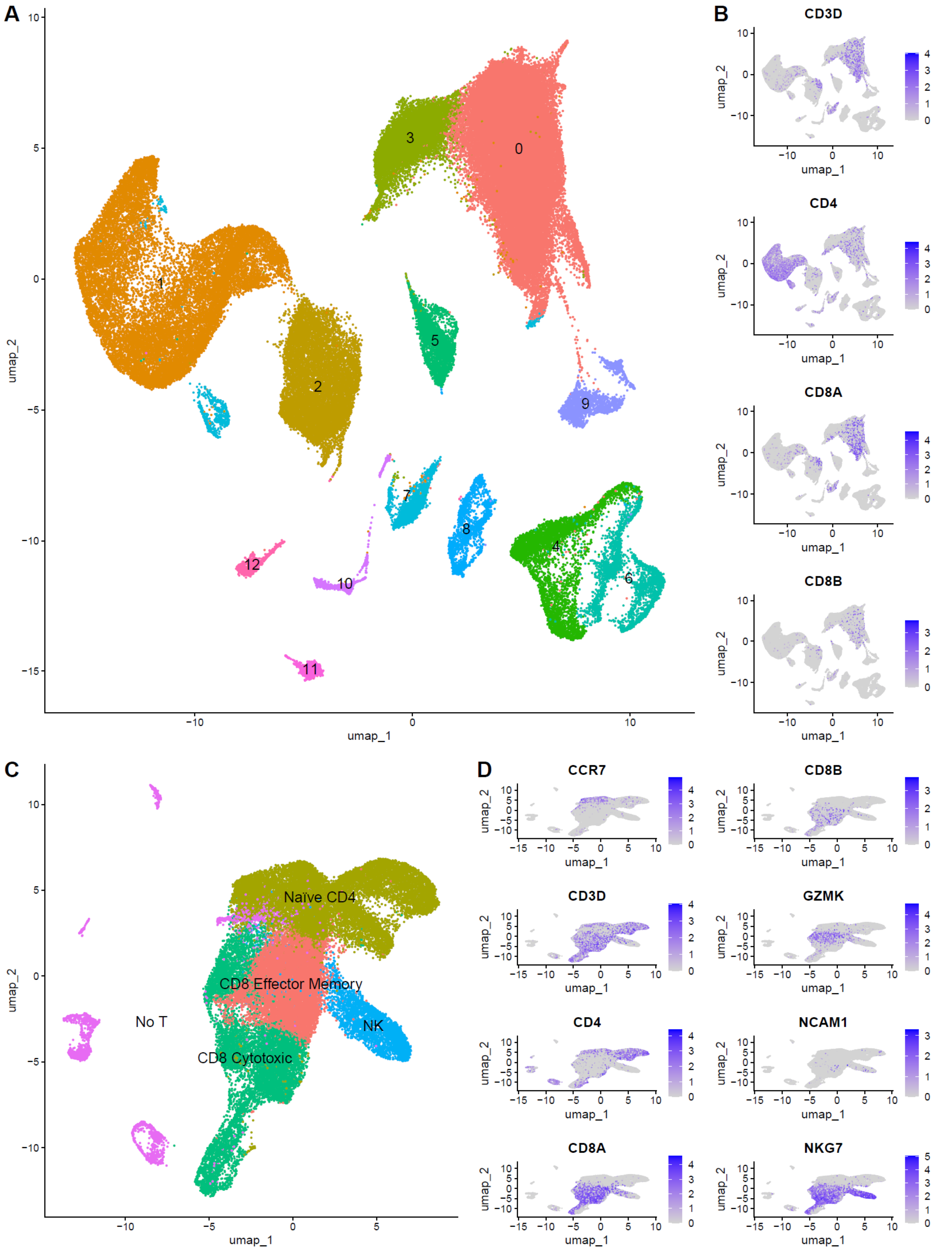

Supplementary Figure S12: **Cell type annotation of an external NSCLC data set [59].** **A** UMAP of the entire NSCLC data set. **B** Expression of major cell type markers used to identify the T cell population. **C** Detailed annotation of the immune cell fraction identified in (B). **D** Markers used to annotate the immune data shown in (C).

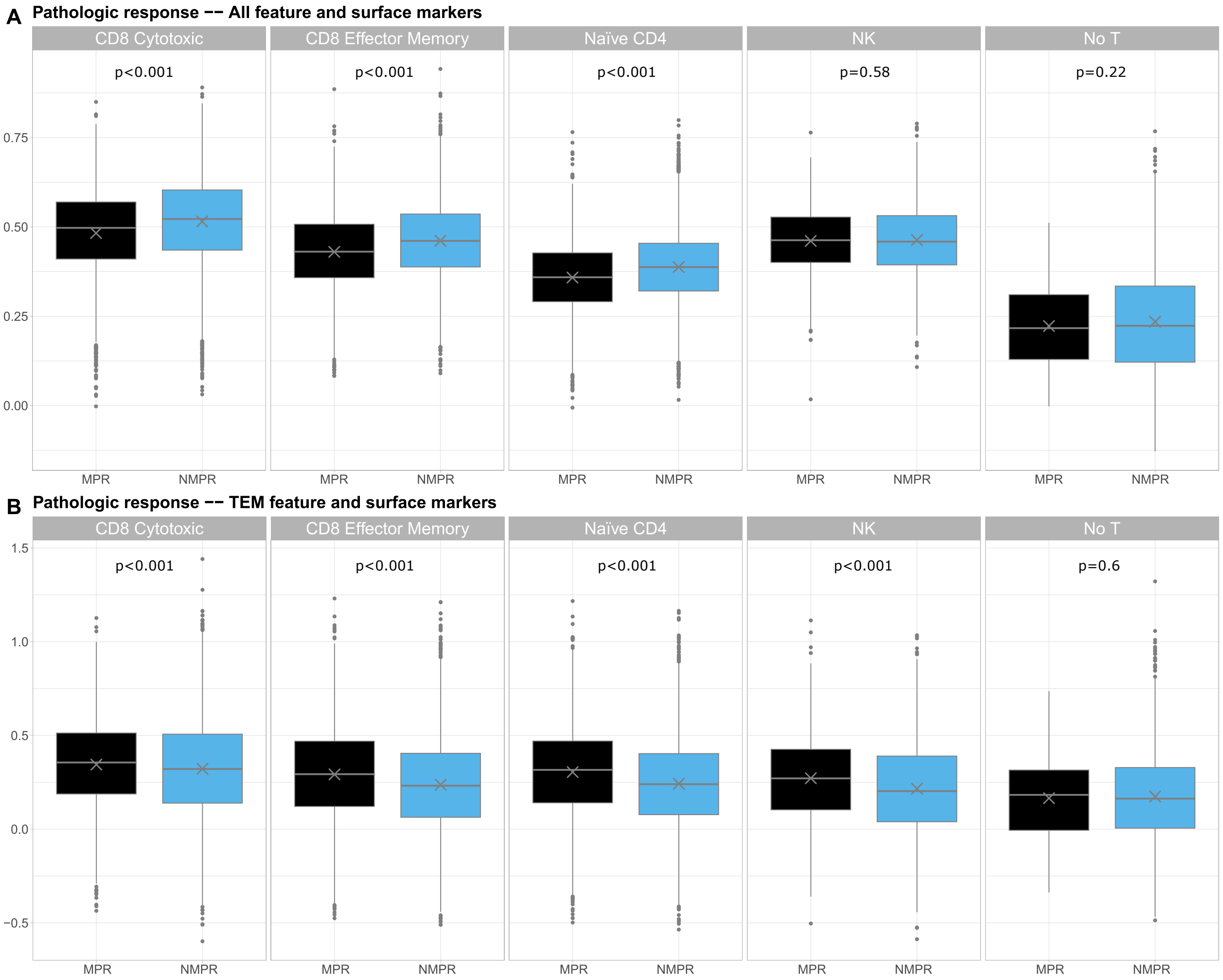

Supplementary Figure S13: **Module Score computation in an external NSCLC data set [59]. A** Module scores using all features in NPR and MPR groups. **B** Module scores using only TEM features in NPR and MPR groups.

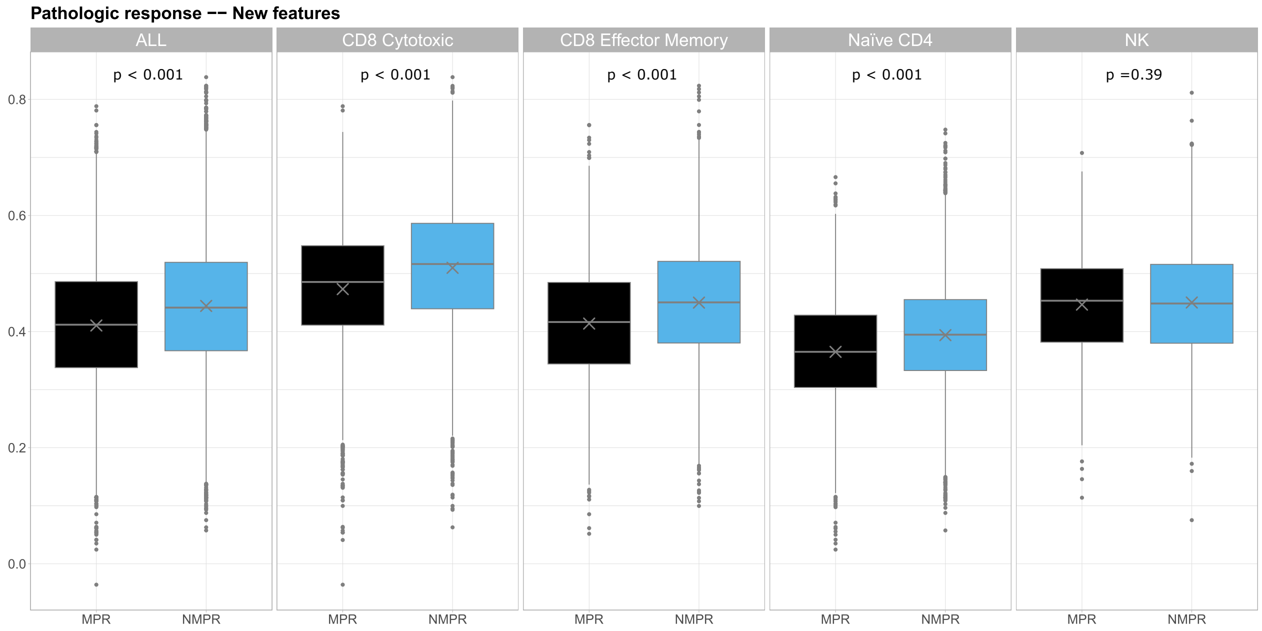

Supplementary Figure S14: **Module Score computation in an external NSCLC data set [59].** Module scores using all features from the general T-cell model in NPR and MPR groups.

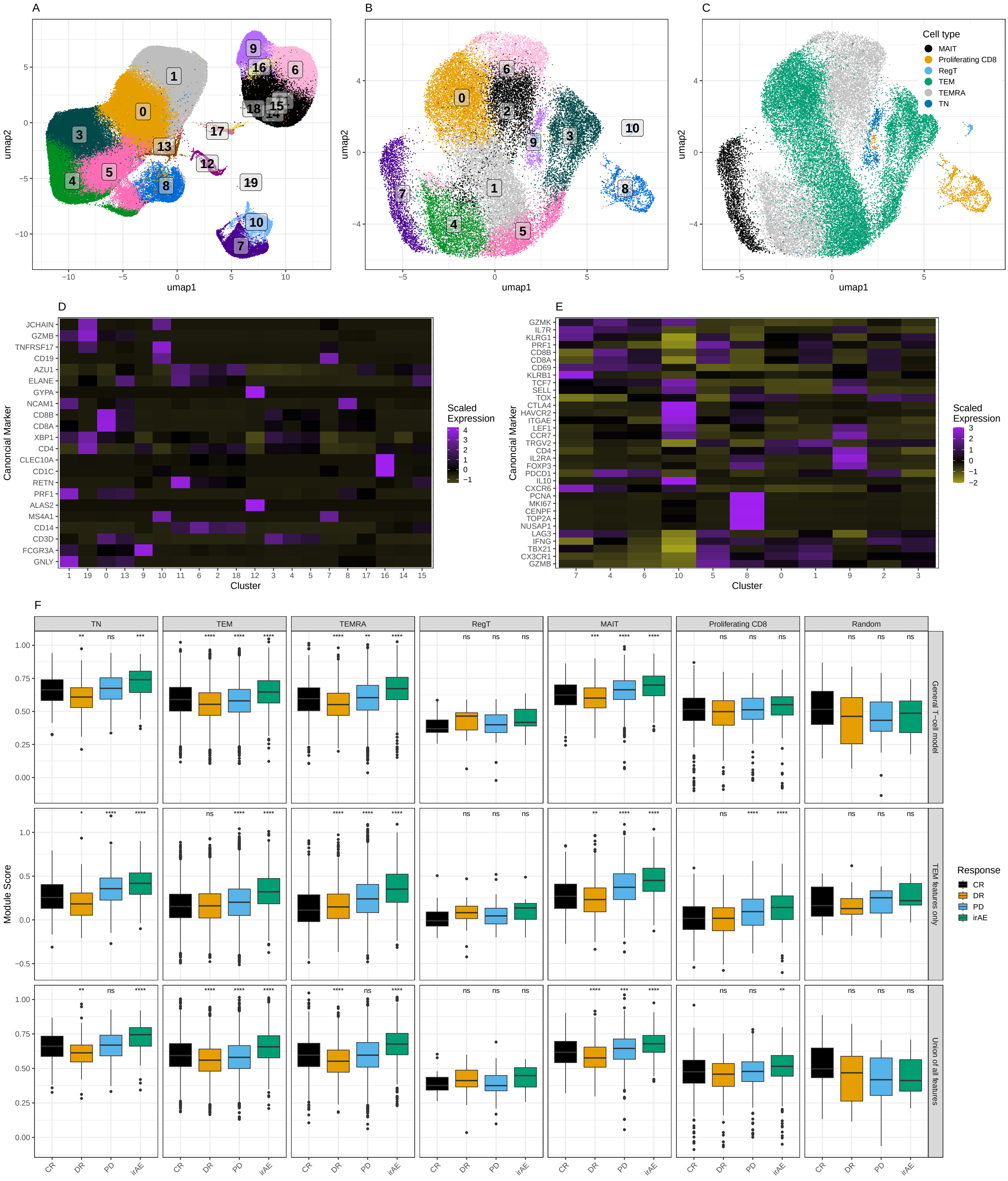

Supplementary Figure S15: **Module Score computation in an external NSCLC data set [33]. A** UMAP of the original PBMC data labeled by cluster numbers. **B** UMAP of the sub clustering for CD8+ T cells (clusters 0 and 13 in A). **C** Same as B but colored by cell type. **D** Pseudo bulk expression of canonical markers to identify major cell types. **E** Pseudo bulk expression of T-cell markers to identify T-cell subtypes. **F** Module scores computed across the T-cell populations for three molecular signatures. Module Scores computed for CR, DP, PD, and irAE patient response classifications.
